# Probiotic characteristics and whole-genome sequence analysis of *Pediococcus acidilactici* isolated from the feces of adult beagles

**DOI:** 10.3389/fmicb.2023.1179953

**Published:** 2023-05-15

**Authors:** Mengdi Zhao, Keyuan Liu, Yuanyuan Zhang, Yueyao Li, Ning Zhou, Guangyu Li

**Affiliations:** ^1^College of Animal Science and Technology, Jilin Agricultural University, Changchun, China; ^2^College of Animal Science and Technology, Qingdao Agricultural University, Qingdao, China; ^3^Shandong Chongzhiyoupin Pet Food Co., Ltd., Weifang, China

**Keywords:** beagles, probiotic, feces, whole-genome sequence, *Pediococcus acidilactici*

## Abstract

The beneficial effects of lactic acid bacteria are well known and recognized as functional foods that are health benefits for companion animals. This study, for the first time, reports the probiotic properties, safety, and whole-genome sequence of *Pediococcus acidilactici* GLP06 isolated from feces of beagles. In this study, candidate probiotic bacteria *P. acidilactici* GLP02 and GLP06 were morphologically characterized and tested for their antimicrobial capacity, tolerance to different conditions (low pH, bile salts, an artificial gastrointestinal model, and high temperature), antibiotic sensitivity, hemolytic activity, cell surface hydrophobicity, autoaggregation activity, and adhesion to Caco-2 cells. *P. acidilactici* GLP06 showed better probiotic potential. Therefore, *P. acidilactici* GLP06 was evaluated for *in vivo* safety in mice and whole-genome sequencing. The results showed, that the supplemented MG06 group (10^10^ cfu/mL), GLP06 was not only nontoxic to mice, but also promoted the development of the immune system, improved resistance to oxidative stress, and increased the diversity of intestinal microorganisms and the abundance of *Lactobacillus*. Whole-genome sequencing showed that *P. acidilactici* GLP06 was 2,014,515 bp and contained 1,976 coding sequences, accounting for 86.12% of the genome, with no drug resistance genes and eight CRISPR sequences. In conclusion, the newly isolated canine-derived *P. acidilactici* GLP06 had good probiotic potential, was nontoxic to mice and promoted the development of immune organs, improved the biodiversity of the intestinal flora, and had no risk of drug-resistant gene transfer, indicating that *P. acidilactici* GLP06 can be used as a potential probiotic for the prevention and treatment of gastrointestinal diseases in companion animals.

## 1. Introduction

Probiotics are defined as “living microorganisms that, when consumed in sufficient quantities, have beneficial effects on the health of the host” (FAO/WHO, 2002; Indian Council of Medical Research Task Force, Co-ordinating Unit ICMR, Co-ordinating Unit DBT, 2011; Guarner et al., 2012). Probiotics have been shown to, affect downstream pathways directly or indirectly, inhibit or activate key signaling pathways such as nuclear factor kappa-B (NFκB) and mitogen-activated protein kinase (MAPK) (Thomas and Versalovic, 2010), enhancing mucosal immune barriers (Jones et al., 2015), and treating abdominal diseases (Preidis and Versalovic, 2009). Lactic acid bacteria (LAB) are known for their safety and are one of the most common types of probiotic microorganisms (Bourdichon et al., 2012). *P. acidilactici* is a type of LAB and belongs to the *Pediococcus* genus. It has been reported to have good probiotic effects, such as reducing constipation and regulating the intestinal flora in mice (Qiao et al., 2021); reducing blood glucose levels and improving pancreatic β-cell function in diabetic rats (Qiao et al., 2021; Widodo et al., 2023); and improving nutrient digestibility and antioxidant capacity in weaned piglets (Dowarah et al., 2018).

The probiotic properties of microorganisms are closely related to host specificity. Therefore, the bacterial species should be derived from the host gut to be successfully used as a probiotic. Unfortunately, most probiotics for companion animals are not initially derived from the canine or feline gastrointestinal tract (GIT) microbiota. Therefore, screening and evaluating strains with more beneficial biochemical potential is still needed. However, the canine and feline GIT are rich in microorganisms with probiotic potential.

Therefore, this study aimed to isolate and identify strains with probiotic potential from beagle feces and to evaluate the probiotic properties of the strains. At the same time, whole-genome sequencing of the strains and mouse dosing tests were conducted to evaluate the safety of the strains. The results of this study will help in the evaluation of microorganisms that could be used as potential strains in the treatment of gastrointestinal disease syndromes to improve companion animal health and welfare.

## 2. Materials and methods

### 2.1. Sampling

Fecal samples were obtained from three healthy adult female beagles from the Jimo Livestock Experimental Farm in Qingdao, China; the test animals had not received antibiotics or probiotics for 2 months prior to sample collection. Samples were collected from the rectum using sterile swabs, quickly placed in sterile test tubes containing 10 mL of Man, Rogosa and Sharpe (MRS) (Haibo, China) and transported under refrigeration to the Animal Nutrition Laboratory of Qingdao Agricultural University.

### 2.2. Isolation and screening of LAB

The collected samples were mixed with a vortex mixer (Scilogex, United States) for 5 min, serially diluted (10^−1^ to 10^−9^), and 0.1 mL of each of the 10^−3^ to 10^−7^ dilutions was applied to MRS agar containing calcium carbonate. Individual colonies with soluble calcium circles were selected for passaging on MRS agar and incubated for 48 h at 37°C. After three passages, the isolates were stored frozen in 50% (w/v) sterile glycerol at −80°C. Supplementary Figure S1 shows the morphological and gram staining results of GLP02 and GLP06.

To identify the isolates, genomic DNA was extracted using a DNA extraction kit (Tsingke, China). Polymerase chain reaction (PCR) amplification was performed using the primers 16S-27F (5’-AGAGTTTGATCCTGGCTCAG-3′) and 16S-1492R (5’-TACGGCTACCTTGTTACGACTT-3′) under the following conditions: pre-denaturation at 94°C for 5 min, denaturation at 94°C for 45 s, annealing at 55°C for 30 s, and extension at 72°C for 45 s for 30 cycles, then extension at 72°C for 10 min. The PCR products were identified by 1.0% agarose gel electrophoresis, and the target bands were recovered with a gel recovery kit (Solarbio, China) and sent to Tsingke Biological Technology for sequencing. A phylogenetic tree was constructed using Mega 11.0 software to determine the evolutionary relationships of the isolated strains.

### 2.3. Functional characteristics

#### 2.3.1. Preparation of cell-free supernatant and bacterial suspension

Selected pure isolates of LAB were inoculated into MRS broth (2% v/v), incubated for 18 h at 37°C, centrifuged at 10,000 rpm for 10 min at 4°C, and the cell-free supernatant (CFS) was collected.

The harvested cells were washed three times with PBS (pH_7.0_) and adjusted to approximately 1 × 10^9^ CFU/mL to obtain a bacterial suspension (BS).

#### 2.3.2. Antimicrobial ability

GLP02 and GLP06 were inoculated into MRS broth and incubated at 37°C for 18 h. The cells were removed by centrifugation at 10,000 rpm for 10 min at 4°C, and the bacterial precipitate (BP) resuspended in PBS (pH_7.0_). Furthermore, indicator bacteria (*E. coli* ATCC25922; *Salmonella* ATCC14028; *Staphylococcus aureus* ATCC25923; *Listeria monocytogenes* ATCC119115; *Pseudomonas aeruginosa* ATCC27853) were inoculated into Lysogeny broth (LB, Haibo, China), incubated at 37°C for 18 h, and the cellular density adjusted to approximately 1 × 10^8^ CFU/mL.

Indicator bacteria were spread on LB agar (Haibo, China) with 6 mm holes punched with Oxford cups: 100 μL of BS, CFS, BP, CFS pH_7.0_, and MRS broth were put inside each well. Finally, the samples were incubated at 37°C for 48 h, and the plates were observed for inhibition circle diameter (IZD) (Argyri et al., 2013; Fontana et al., 2015). Each test was repeated in triplicate.

#### 2.3.3. Growth and acid-producing ability

An inoculum of 2.0% (v/v) of each strain was cultured in MRS broth, incubated at 37°C for 48 h. At 1, 2, 3, 5, 7, 9, 12, 15, 18, 21, 24, 27, 30, 33, 36, and 48 h, the absorbance OD_600_ and pH of the MRS were measured and used to plot the growth and acid-production curves.

#### 2.3.4. Growth curves at different pH

The isolated strains were added at 2% (v/v) inoculum to MRS liquid at initial pH 1.0, 2.0, 3.0, 4.0, 5.0, 6.0, and 7.0 and incubated at 37°C. The OD_600_ absorbance was measured at 3, 6, 9, 12, 18, 24, 30, 36, and 42 h, respectively, and the growth curves were plotted.

#### 2.3.5. Tolerance to gastrointestinal conditions

##### 2.3.5.1. Acid and bile salt resistance

The cultures were inoculated into MRS broth, incubated at 37°C for 18 h, washed twice with PBS (pH_7.0_), and resuspended in PBS (pH_2.5_) at 37°C for 3 h. Samples were collected at 0 and 3 h after incubation and analyzed for cell counts (Yu et al., 2020).

The cultures were inoculated into MRS broth, incubated at 37°C for 18 h, washed twice with PBS (pH_7.0_), and resuspended in 0.3 and 0.5% bile salts (Solarbio, China) at 37°C for 4 h. At 0 and 4 h after incubation, samples were collected and CFU counts were determined.

##### 2.3.5.2. Resistance to artificial gastrointestinal models

Samples of the strains were collected during the stable growth period, centrifuged at 10,000 rpm for 10 min at 4°C, washed twice with PBS (pH_7.0_), resuspended in gastric juice, and incubated at 37°C for 1.5 h. Following this, they were centrifuged again, washed twice with PBS (pH_7.0_), and resuspended in intestinal fluid at 37°C for 2 h (Zárate et al., 2000). Each test was repeated in triplicate. Survivability was calculated using the formula:


(1) $$\mathrm{Survivability}\;\left(\%\right)=T_{\mathrm{treatment}}{}^{1}/T_{\mathrm{initial}}{}^{1}\times 100\%$$

(1) *T*_initial_^1^ and *T*_treatment_^1^ are the numbers of surviving bacteria (log CFU/mL) before and after treatment, respectively.

#### 2.3.6. Tolerance to high temperature

The strains were incubated overnight and placed in water baths at temperatures of 50°C, 60°C, 70°C, 80°C, and 90°C for 5 min (Chen et al., 2020). Survivability was calculated using the formula:


(2) $$\mathrm{Survivability}\;\left(\%\right)=T_{\mathrm{treatment}}{}^{2}/T_{\mathrm{initial}}{}^{2}\times 100\%$$

(2) *T*_initial_^2^ and *T*_treatment_^2^ are the numbers of viable bacteria at 0 min and 5 min, respectively (log CFU/mL).

#### 2.3.7. Safety assessment of the isolated strains

##### 2.3.7.1. Hemolytic activity

The cultures were incubated overnight (18 h), streaked onto blood agar containing 5% (v/v) sheep blood (Oxoid, Germany) and incubated for 48 h at 37°C (Reuben et al., 2019). *Staphylococcus aureus* (ATCC25923) served as a positive control.

##### 2.3.7.2. Antibiotic susceptibility

Based on recommendations for evaluating the safety of probiotics, 21 antibiotics were selected for testing in the form of 6 mm paper tablets (BIO-KONT, China): penicillin, oxacillin, ampicillin, piperacillin, imipenem, vancomycin, streptomycin, gentamicin, amikacin, kanamycin, tetracycline, chloramphenicol, minocycline, doxycycline, cotrimoxazole, azithromycin, erythromycin, clindamycin, norfloxacin, ciprofloxacin, and levofloxacin.

Colonies of the isolates were inoculated into PBS (pH_7.0_) to obtain 0.5 McFarland turbid cultures. Antibiotic-containing paper sheets were dispensed onto MRS agar medium coated with 0.5 McFarland and cultivated for 48 h at 37°C (Mancini et al., 2020). The IZD (including the disk diameter) was measured, and isolates were categorized as sensitive (≥ 21 mm), intermediate (16–20 mm), or resistant (≤ 15 mm) (Abbasiliasi et al., 2012).

#### 2.3.8. Cell surface hydrophobicity

The strains were collected during the stable growth period, centrifuged at 10,000 rpm for 10 min at 4°C, washed three times with PBS (pH_7.0_), and resuspended in PBS (pH_7.0_) to an OD_600_ of about 0.25 ± 0.05 (A_1_) in order to standardize. Subsequently, an equal volume of xylene and chloroform was added and mixed by vortexing for 90 s (Collado et al., 2008). The aqueous phase was removed after 3 h of incubation at 37°C and its absorbance at 600 nm was measured (A_2_). The cell surface hydrophobicity was calculated using the formula:


(3) $$\mathrm{Cell Surface Hydrophobicity}\;\left(\%\right)=\left(1-A_{2}/A_{1}\right)\times 100\%$$

(3) *A*_1_ and *A*_2_ are the absorbances at 0 h and 3 h, respectively.

#### 2.3.9. Autoaggregation activity

An overnight culture was washed three times with PBS (pH_7.0_), the OD_600_ was measured (A_3_) and the sample mixed for 15 s by vortexing (Hernández-Alcántara et al., 2018). The culture was removed after 8 h of incubation at 37°C and its absorbance at 600 nm was measured (A_4_). The autoaggregation activity was calculated using the formula:


(4) $$\mathrm{Auto aggregation activity}\;\left(\%\right)=\left(1-A_{4}/A_{3}\right)\times 100\%$$

(4) *A*_3_ and *A*_4_ are the absorbances at 0 h and 8 h, respectively.

#### 2.3.10. Adhesion to human colon carcinoma (Caco-2) cells

The adhesion ability of LAB strains was evaluated using Caco-2 cells. Caco-2 cells were cultured using minimal essential medium (MEM) supplemented with 20% (v/v) fetal bovine serum (Gibco, United States), 100 μg penicillin, and 100 μg streptomycin (Sigma-Aldrich, United States).

Caco-2 cells with good growth and 90% wall adherence were digested with 0.25% trypsin–EDTA, incubated in 24-well plates at 37°C until the cells grew to a monolayer, washed the cultured cells three times with PBS (pH_7.0_), and 1 mL of MEM complete culture solution without double antibiotics and serum added. The bacterial solution was washed three times with PBS (pH _7.0_). The bacterial solution was resuspended in PBS (pH _7.0_) and adjusted to 1 × 10^7^ CFU/mL, and 1 mL of the bacterial suspension added to each well, followed by incubation for 1.5 h at 37°C in the presence of 5% CO_2_. Following this, 1.0% Triton X-100 solution (Solarbio, China) was added to the bacterial cells for 15 min (Cheon et al., 2020). The cell solution was recovered at 8000 rpm for 10 min, and resuspended in PBS (pH _7.0_), and wells without Caco-2 cells to which only bacterial solution was added served as blank controls (A_5_), and the number of bacteria adhering to Caco-2 cells (A_6_) was assessed by serially dilution of the bacterial cytosol and incubating at 37°C for 24–48 h.


(5) $$\mathrm{Adhesion rate}\;\left(\%\right)=\left(A_{6}/A_{5}\right)\times 100\%$$

(5) A_5_ and A_6_ represent the viable bacterial cell number before treatment (log CFU/mL) and after treatment (log CFU/mL), respectively.

#### 2.3.11. *In vitro* analysis of antioxidant activity

##### 2.3.11.1. The ability to scavenge 2,2-diphenyl-1-picrylhydrazyl radicals

A DPPH reagent test kit (Solarbio, China) was used to measure the DPPH radical scavenging ability of the isolated strains. The absorbance of the resulting solution was measured at 517 nm. Ascorbic acid was used as a positive control. The DPPH radical scavenging rate was calculated as follows:


(6) $$\begin{array}{l} \mathrm{DPPH radical scavenging rate}\%\\ =\left[\frac{\left[A_{blank}{}^{1}-\left(\;A_{assay}{}^{1}-A_{control}{}^{1}\right)\right]}{A_{blank}{}^{1}}\right]\times 100\% \end{array}$$

(6) A_assay_^1^ is the absorbance of the sample to be tested; A_control_^1^ is the absorbance of a mixture of the supernatant of the isolate treatment and anhydrous ethanol; A_blank_^1^ is the absorbance of a mixture of the extract and working solution.

##### 2.3.11.2. The ability to scavenge 3-ethylbenzthiazoline-6-sulfonic acid (ABTS) radicals

The ABTS radical scavenging ability of the isolated strains was measured by an ABTS reagent test kit (Solarbio, China). The absorbance of the resulting solution was measured at 405 nm. Ascorbic acid was used as a positive control. The ABTS radical scavenging rate was also calculated using Formula (6).

### 2.4. Safety evaluation of the strain in mice

#### 2.4.1. Animal experiments and sample collection

Eighty Kunming mice (4-weeks-old) were randomly divided into four groups, half males and half females. LG06 group (10^9^ CFU/mL), MG06 group (10^10^ CFU/mL), and HG06 group (10^11^ CFU/mL) mice were orally administered 0.2 mL of different concentrations of bacterial solution, while CK group mice were given an equal volume of saline. Every 3 days, body weight and food intake were measured, and the health status of the mice was recorded. After 27 days, the mice were fasted for 12 h and anesthetized with 1% sodium pentobarbital (50 mg/kg). Blood was collected from the abdominal aorta, and serum was collected by centrifugation (3,500 rpm, 4°C, 10 min) for analysis. After execution, the mice in each group were dissected and the viscera observed, and organs were collected and weighed. The organ coefficient was calculated as organ weight/body weight ×100 (Li et al., 2019).

#### 2.4.2. Serum biochemical parameters in mice

Liver function, kidney function index, and antioxidant parameters were measured in mouse serum using commercial enzyme linked immunosorbent assay (ELISA) kits (Jiancheng, China). The data were measured by an enzyme marker or biochemical autoanalyzer (Selectra E, The Netherlands).

#### 2.4.3. Gut microbiota in mice

Before the end of the experiment, feces were randomly collected from 10 mice in each group and stored at −80°C. DNA was extracted from the feces samples, and the V_3_–V_4_ region of the bacterial 16S rRNA gene was sequenced using the Illumina NovaSeq platform. The sequences were clustered into Operational Taxonomic Units (OTUs) with 97% similarity. All analyzes from clustering to determining α and β diversity were performed using QIIME. The data visualization web address is https://www.chiplot.online/.

### 2.5. Whole-genome sequencing

The genome was sequenced on the PacBio and Nanopore platforms at Allwegene Technology Co. (Beijing, China) and reads were assembled using Unicycler (Version: 0.5.0,^1^) (Wick et al., 2017). The genes were analyzed with Clusters of Orthologous Groups (COG) (Tatusov et al., 2000), Kyoto Encyclopedia of Genes and Genomes (KEGG) (Kanehisa et al., 2004), UniProt (Apweiler et al., 2004), and Gene Ontology (GO) (Ashburner et al., 2000). Clustered regularly interspersed short palindromic repeats (CRISPR) were identified by MinCED (Version: 0.4.2,^2^). To confirm the presence of resistance genes, all identified coding sequences (CDS) were compared against the Comprehensive Antibiotic Resistance Database (CARD) (Alcock et al., 2020).

### 2.6. Statistical analysis

Data were expressed as mean ± standard deviation (SD) and analyzed using the *t*-test and one-way and two-way ANOVA in GraphPad Prism 8.0, with significant differences between groups at *p* < 0.05.

## 3. Results

### 3.1. Antimicrobial ability

**Figure 1:**
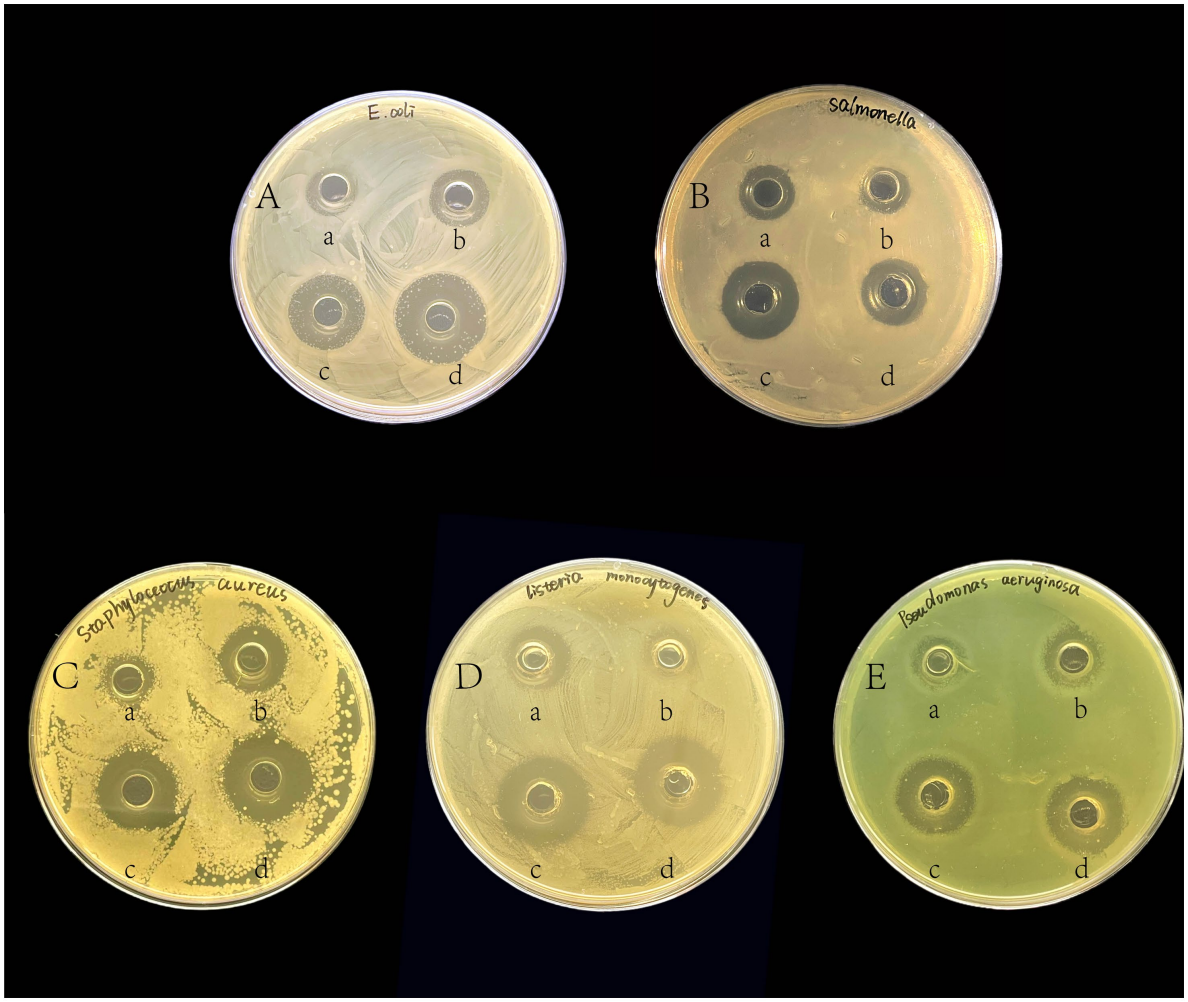
The inhibitory effects of isolated strains against pathogenic indicator bacteria. The pathogenic indicator bacteria were **(A)** *E. coli*; **(B)** *Salmonella*; **(C)** *S. aureus*; **(D)** *L. monocytogenes*; and **(E)** *P. aeruginosa.* In each LB agar plate, (a) was added to the cell-free supernatant of GLP02; (b) was added to the cell-free supernatant of GLP06; (c) was added to the bacterial suspension of GLP02; (d) was added to the bacterial suspension of GLP06.

From all the strains isolated, the two most effective strains, GLP02 and GLP06, were selected for further study. Figure 1 shows the inhibitory ability of GLP02 and GLP06 strains against pathogenic indicator bacteria. The inhibitory effect of CFS GLP06 on *E. coli*, *S. aureus* and *P. aeruginosa* was significantly higher than that of CFS GLP02 (*p* < 0.05), but its inhibition of *L. monocytogenes* was significantly lower than that of CFS GLP02 (*p* < 0.05). BS GLP02 showed significantly more inhibitory ability on *Salmonella* than GLP06 (*p* < 0.05), while the inhibition results for other pathogenic bacteria were not significantly different (*p* > 0.05). Overall, the inhibitory ability of BS was significantly higher than that of CFS (*p* < 0.05). The results indicated that GLP02 and GLP06 had better inhibition of pathogenic bacteria. Supplementary Figure S2 demonstrates the inhibitory effect of BP, MRS broth and CFS pH 7.0 on pathogenic bacteria.

### 3.2. Identification of GLP02 and GLP06

**Figure 2:**
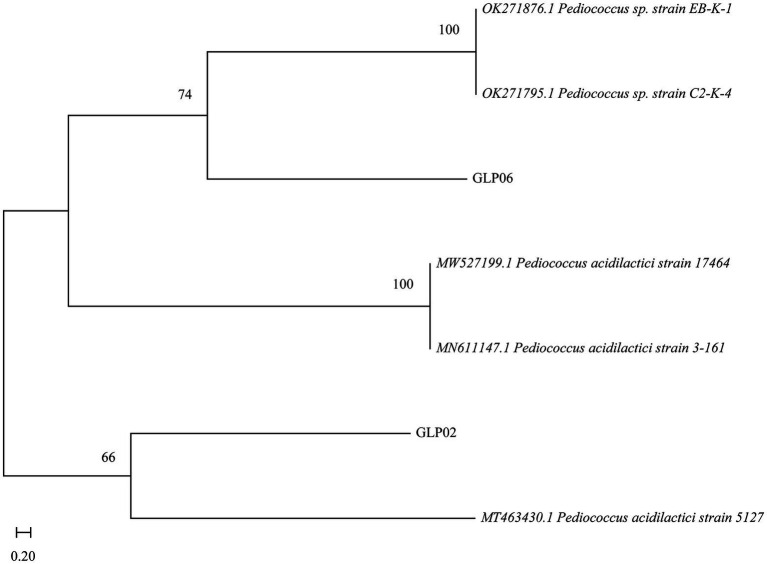
Neighbor-joining phylogenetic tree of GLP02 and GLP06.

To identify the species of the isolates, a molecular phylogenetic analysis was constructed using the 16S sequencing results. The results showed that GLP02 and GLP06 were *P. acidilactici* (Figure 2). Whole-genome sequencing of GLP06 also showed the same result (Supplementary Table S3).

### 3.3. Functional characteristics of GLP02 and GLP06

#### 3.3.1. Growth, acid-producing ability, and growth curves at different pHs

**Figure 3:**
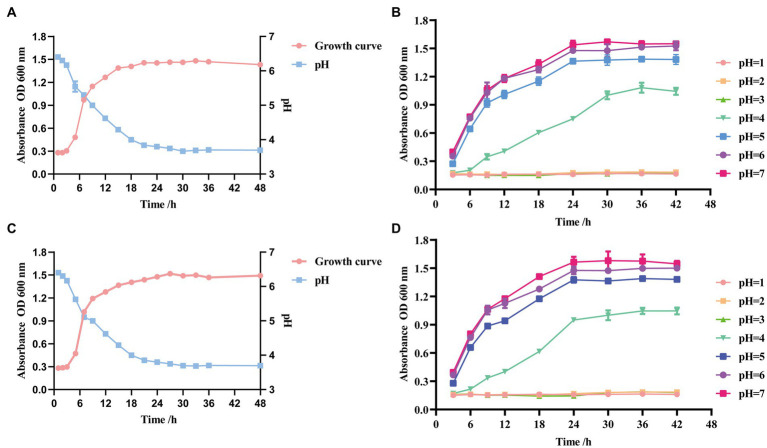
Growth, acid-producing ability and growth curves at different pH of selected strains. **(A)** Growth and acid-producing curve of GLP02; **(B)** Growth curves at different pH of GLP02; **(C)** Growth and acid-producing curve of GLP06; and **(D)** Growth curves at different pH of GLP06.

The growth of the two strains was slow from 0 to 3 h after inoculation, in the growth stagnation period. After 3 h, the OD_600_ increased rapidly, indicating the beginning of the logarithmic growth period. After 18 h, the OD_600_ leveled off and entered a stable period. The trend of pH was generally consistent with the growth curve. GLP02 was stable at approximately 3.67 (Figure 3A), and GLP06 was stable at approximately 3.68 (Figure 3C).

The growth of strains GLP02 (Figure 3B) and GLP06 (Figure 3D) was entirely inhibited at pH ≤3.0. The strains could grow slowly at pH_4.0_ and normally at pH_5.0_, pH_6.0_ and pH_7.0._ However, there were some differences in the OD_600_ when reaching the stable phase.

#### 3.3.2. Tolerance to different conditions

**Figure 4:**
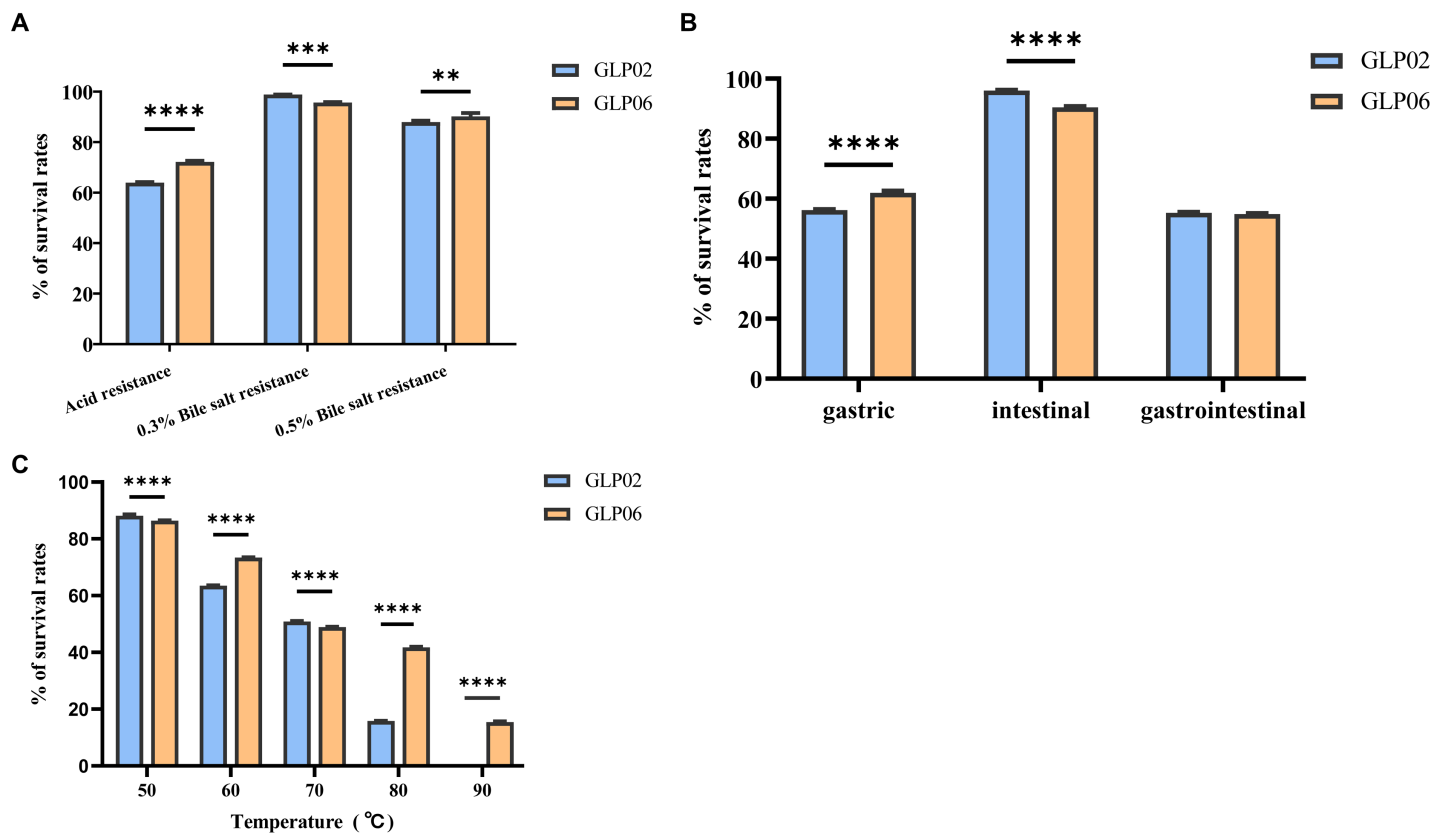
Survival rates of isolated strains after exposure to different conditions. **(A)** Acid and bile salt resistance; **(B)** Artificial gastric juice, intestinal fluid, and gastrointestinal tract models; and **(C)** High temperature. Values were displayed as the mean ± SD, **p < 0.01, ***p < 0.001 and ****p < 0.0001 indicate differences between two *P. acidilactici* strains.

Figure 4 displays the survival rates for acid, bile salt resistance, gastrointestinal models, and high temperature. GLP06 (72.17%) showed significantly higher survival rates than GLP02 (63.97%) at pH_2.5_ (*p* < 0.0001). The graph shows that there was a slight fall in the survival rates of GLP02 and GLP06 at 0.3% bile salt, although GLP02 (98.84%) showed significantly higher resistance than GLP06 (95.70%, *p* < 0.001). However, GLP06 (90.22%) showed significantly higher resistance to 0.5% bile salt than GLP02 (87.95%, *p* < 0.01). The study demonstrated the good viability of GLP02 and GLP06 under both gastric and intestinal conditions, with mean viability rates of 55.32 and 54.83%, respectively. Treatment of *P. acidilactici* GLP06 at 50, 60, and 70°C for 5 min had little effect on its survival, but after 5 min at 80 and 90°C, the % survival of GLP02 and GLP06 decreased significantly. The higher the temperature above 70°C, the greater the damage to the bacteria, although GLP06 had better resistance than GLP02, as shown in Figure 4.

#### 3.3.3. Safety assessment of GLP02 and GLP06

##### 3.3.3.1. Hemolytic activity

GLP02 and GLP06 did not present any hemolysis (called *γ*-hemolysis) and can be generally accepted as safe; the data are shown Supplementary Figure S4.

##### 3.3.3.2. Antimicrobial susceptibility

To ensure safety, the phenotypic antibiotic susceptibility of GLP06 was investigated against 21 antibiotics. It can be seen from the data in Table 1 that GLP06 was susceptible to piperacillin, imipenem, chloramphenicol, and erythromycin, and showed intermediate susceptibility to clindamycin, doxycycline, and levofloxacin, but was resistant to the rest of the antibiotics. Data for GLP02 are shown in Supplementary Table S5.

**Table 1:** Antibiotic resistance of strain GLP06.

Antimicrobial classes	Antimicrobial agents	Disk dose (μg)	Inhibition zone diameters/mm (IZD)^a^		
			≤15 mm (R)	16–20 mm (I)	≥21 mm (S)
β-lactams antibiotics	Penicillin	10	X^R^		
	Oxacillin	1	X^R^		
	Ampicillin	10	X^R^		
	Piperacillin	100			22.13 ± 3.75^S^
	Imipenem	10			26.15 ± 1.18^S^
Glycopeptides	Vancomycin	30	X^R^		
Aminoglycosides antibiotics	Streptomycin	10	X^R^		
	Gentamicin	10	X^R^		
	Amikacin	30	X^R^		
	Kanamycin	30	X^R^		
Broad-spectrum antibiotics	Tetracycline	30	X^R^		
	Chloramphenicol	30			23.22 ± 3.89^S^
	Minocycline	30	X^R^		
	Doxycycline	30		16.13 ± 3.76^I^	
	Cotrimoxazole	25	X^R^		
Macrolides	Azithromycin	15	X^R^		
	Erythromycin	15			21.72 ± 1.25^S^
	Clindamycin	2		18.25 ± 3.79^I^	
Fluoroquinolone antibiotics	Norfloxacin	10	X^R^		
	Ciprofloxacin	5	X^R^		
	Levofloxacin	5		15.47 ± 0.96^I^	

a R, Resistant; I, Intermediate; S, Sensitive; X, No inhibition zone observed. Values are mean with SD of three replications.

#### 3.3.4. Cell surface hydrophobicity and autoaggregation activity

**Figure 5:**
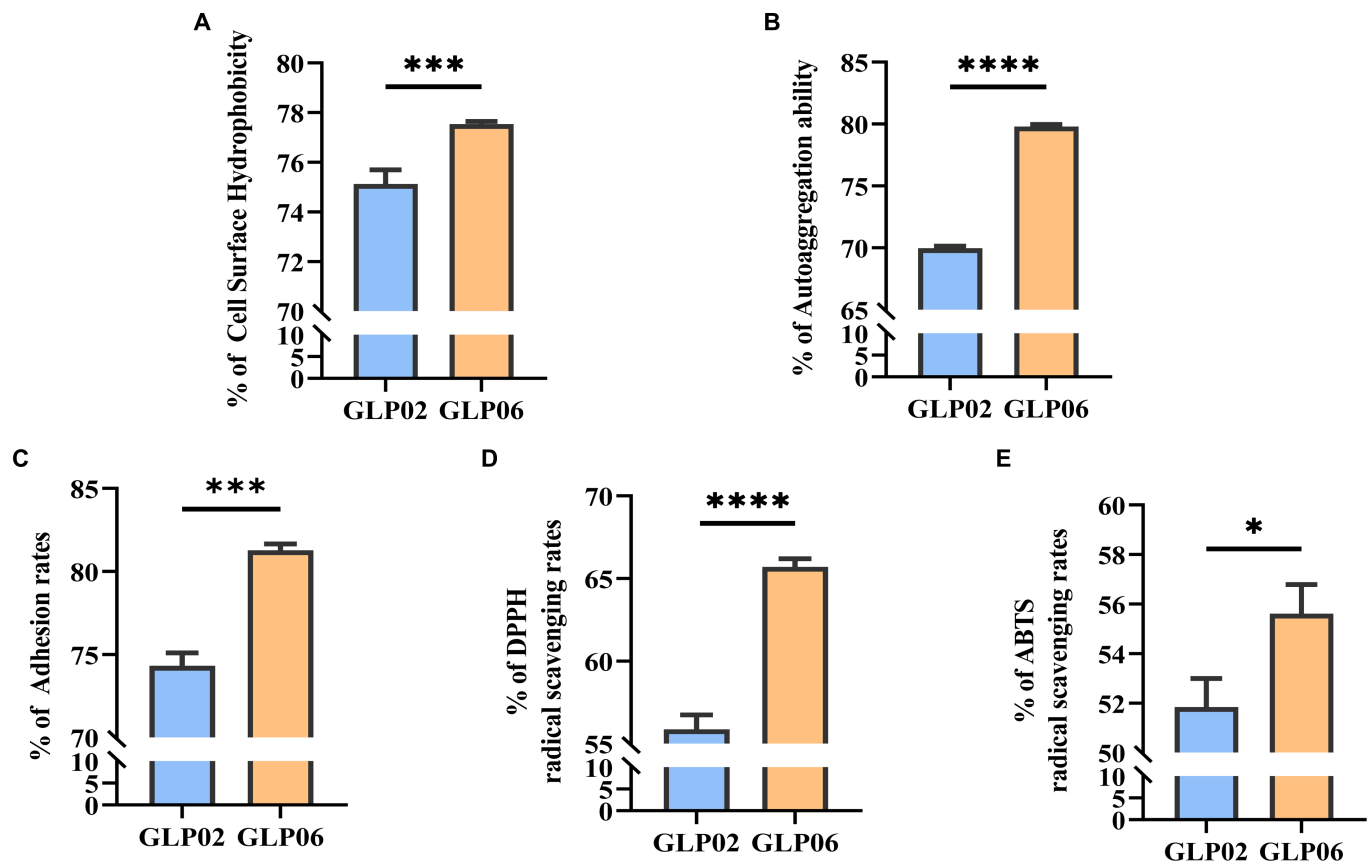
Cell surface hydrophobicity, auto aggregation ability, adhesion, and antioxidant activity of *P. acidilactici* strains. **(A)** Cell surface hydrophobicity of *P. acidilactici* strains; **(B)** Auto aggregation ability of *P. acidilactici* strains; **(C)** Adhesion of *P. acidilactici* strains to Caco-2 cells; **(D)** DPPH radical scavenging rate of *P. acidilactici* strains; and **(E)** ABTS radical scavenging rate of *P. acidilactici* strains. Values displayed are the mean ± SD, *p < 0.05, ***p < 0.001 and ****p < 0.0001 indicate differences between the two *P. acidilactici* strains.

Figures 5A,B show that the two strains had high CSH and autoaggregation. GLP06 (77.54%) had significantly higher % CSH than GLP02 (74.89%, *p <* 0.001). The autoaggregation of GLP02 (63.97%) was significantly lower than that of GLP06 (79.80%, *p <* 0.0001).

#### 3.3.5. Adhesion to Caco-2 cells

The two strains showed a high level of adhesion ability to Caco-2 cells (Figure 5C). GLP06 (81.27%) showed significantly higher % adhesion than GLP02 (74.31%, *p <* 0.001).

#### 3.3.6. *In vitro* analysis of antioxidant activity

Figures 5D,E show the antioxidant activity of the two strains. GLP06 had significantly more antioxidant ability for DPPH (*p* < 0.0001) and ABTS (*p* < 0.05) than GLP02. The two strains showed high antioxidant ability.

### 3.4. Safety evaluation of the GLP06 strain in mice

#### 3.4.1. Effect of GLP06 on growth performance and organ coefficients in mice

**Figure 6:**
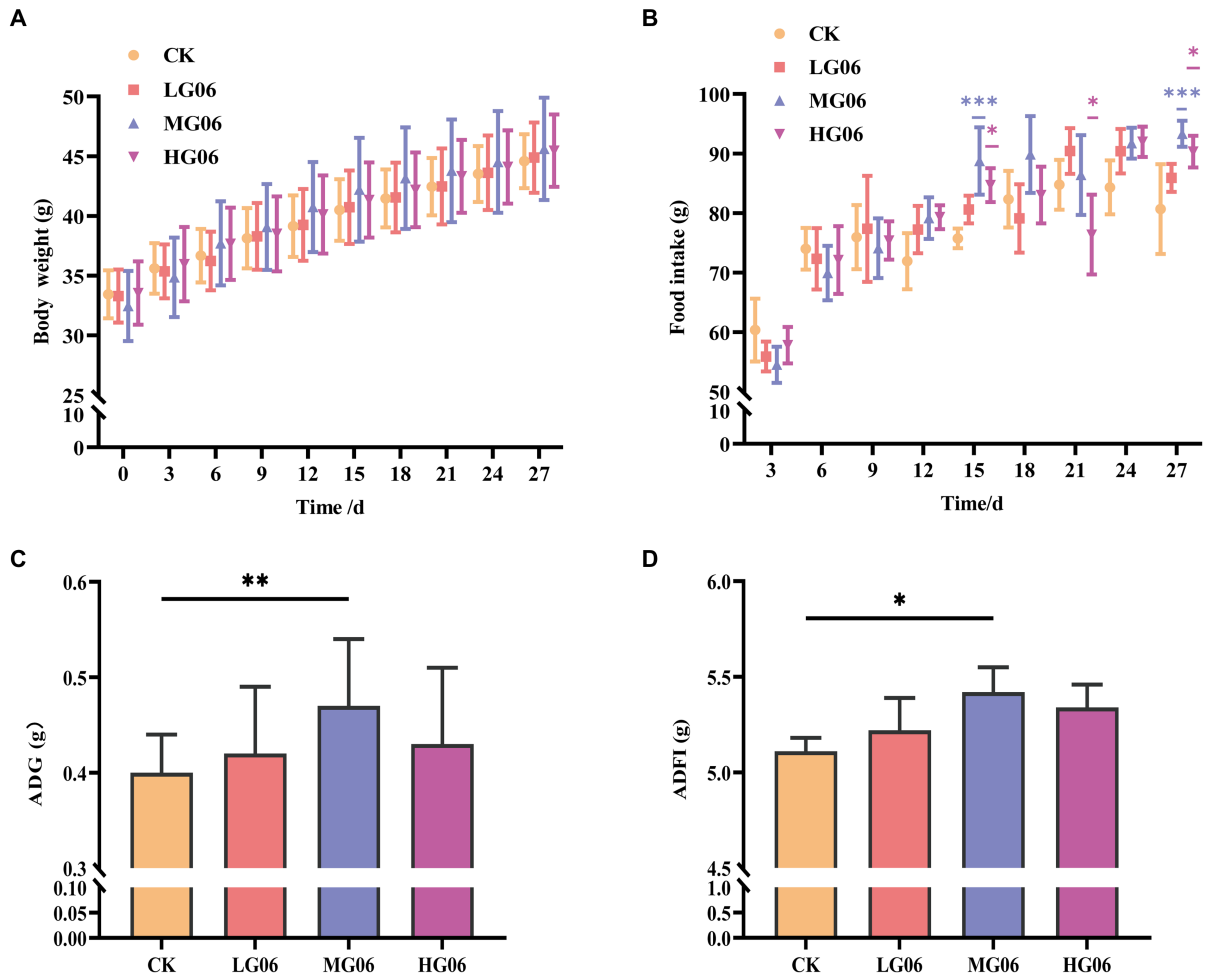
Effect of GLP06 supplementation on body weight and food intake in mice. **(A)** Body weight; and **(B)** Food intake were measured every 3 days; and **(C)** Average daily gain and **(D)** Average daily food intake were calculated at the end of the trial period. Values were displayed as the mean ± SD. *p < 0.05, **p < 0.01, and ***p < 0.001 indicate differences among the four groups, n = 10.

We evaluated the effects of GLP06 supplementation on body weight and food intake in mice. The body weights of mice supplemented with different concentrations of GLP06 were not significantly different from those of the CK group, and the body weight of mice gradually increased during the trial (Figures 6A,B). In addition, the average daily gain (ADG) and average daily food intake (ADFI) of mice in the MG06 group were significantly higher than that of the CK group (*p* < 0.01 or *p* < 0.05; Figures 6C,D).

**Figure 7:**
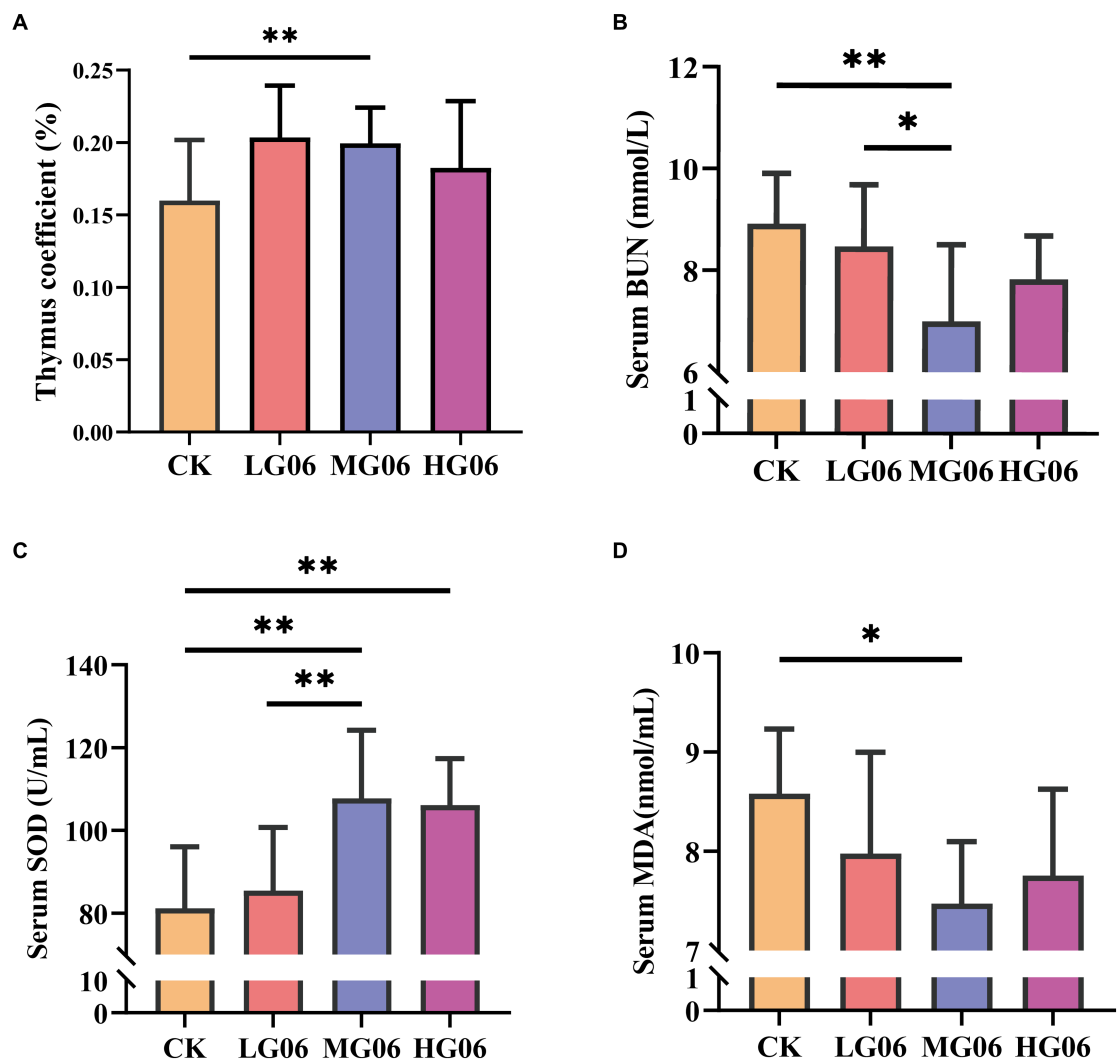
Effect of GLP06 supplementation on organ coefficients and serum biochemical parameters in mice. **(A)** Thymus coefficient; **(B)** Blood urea nitrogen; **(C)** Superoxide dismutase and **(D)** Malondialdehyde were determined by use of commercial ELISA kits. Values were displayed as the mean ± SD. *p < 0.05 and **p < 0.01 indicate differences among the four groups, *n* = 10.

Next, we investigated how GLP06 affected organ coefficients in mice. The organ weights of the heart, liver, spleen, and kidneys were not significantly different from those of the CK group (Supplementary Figures S6A–D). It is worth noting that the thymus organ coefficients were significantly higher than in the CK group (*p* < 0.01; Figure 7A).

#### 3.4.2. Effect of GLP06 on antioxidant and liver performance in mice

We also investigated how GLP06 affected blood markers in mice. The serum aspartate aminotransferase (AST), alanine aminotransferase (ALT), total bilirubin (T-BIL), indirect bilirubin (I-BIL), and direct bilirubin (D-BIL) concentrations of mice supplemented with GLP06 were not statistically significantly different from those of the CK group (Supplementary Figures S7A–E). At the same time, the blood urea nitrogen (BUN) levels in the MG06 group were significantly lower than those of the CK group and LG06 group (*p* < 0.05 or *p* < 0.01; Figure 7B).

The superoxide dismutase (SOD) levels of mice in the HG06 and MG06 groups were significantly higher than those of the LG06 and CK groups (*p* < 0.01; Figure 7C). In addition, the malondialdehyde (MDA) levels of mice in the MG06 group were significantly lower than those of the CK group (*p* < 0.05; Figure 7D).

#### 3.4.3. Effect of GLP06 on gut microbiota in mice

**Figure 8:**
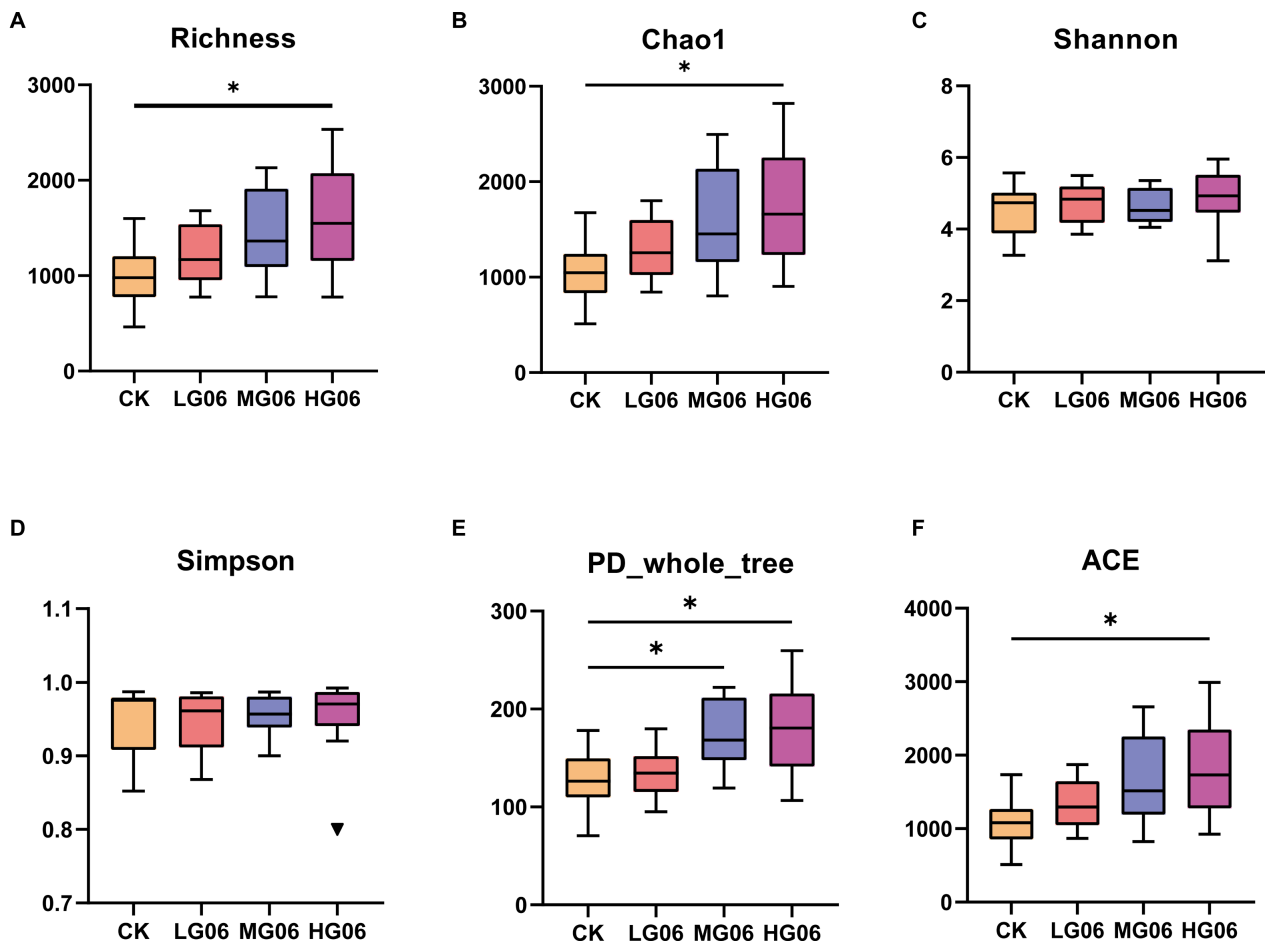
Effects of GLP06 supplementation on alpha diversity of intestinal microflora in mice. Box plot depicting **(A)** Richness; **(B)** Chao1; **(C)** Shannon; **(D)** Simpson; **(E)** PD-whole-tree; and **(F)** ACE of intestinal microflora in mice. *p < 0.05 indicate differences among the four groups, *n* = 10.

Alpha diversity can reflect the abundance and diversity of microbial communities. The richness, Chao1, and ACE indices of the intestinal microbes in the HG06 group were significantly higher than those of the other groups (*p* < 0.05; Figures 8A,B,F), and PD whole tree values in the HG06 and MG06 group were significantly higher than in the CK group (*p* < 0.05; Figure 8E). In addition, Shannon and Simpson indices in the mice supplemented with GLP06 showed no significant difference from those of the CK group (Figures 8C,D).

**Figure 9:**
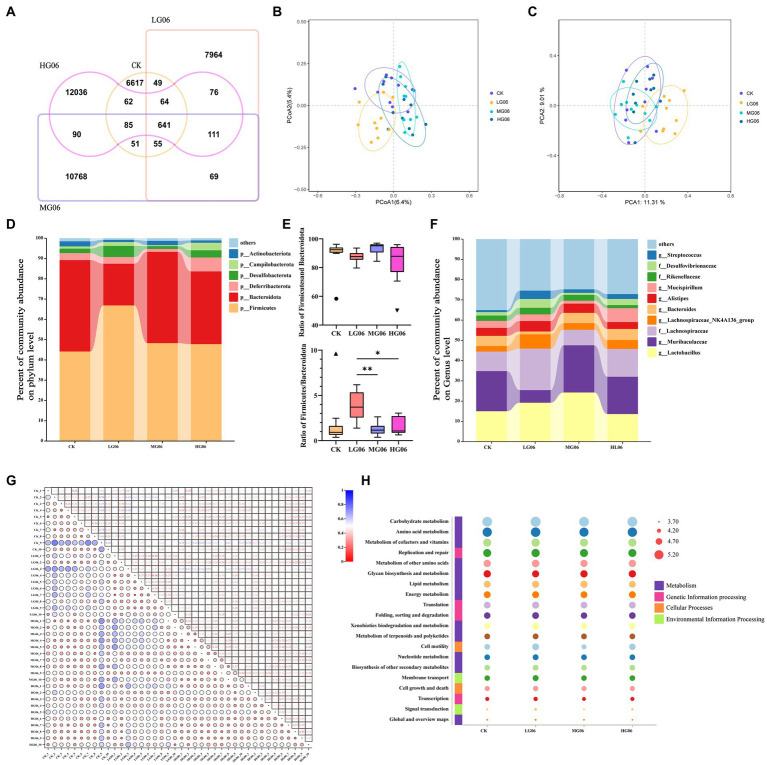
Effects of GLP06 supplementation on the intestinal microflora of mice. **(A)** Veen; **(B)** Principal Co-ordinates Analysis; **(C)** Principal Component Analysis; **(D)** Community abundance on phylum level of the top 6; **(E)** Community abundance on phylum level of *Bacteroidota* and *Firmicutes* **(F)** Community abundance on genus level of the top 10; **(G)** Based on the UNIFRAC heatmap map and **(H)** analysis of the top 20 KEGG pathways were presented as bubble plots. *p < 0.05, **p < 0.01 indicate differences among the four groups, *n* = 10.

Figure 9A shows the number of common and unique ASV in each group, and the number of ASV increases with the increase of GLP06 concentration. Principal co-ordinates analysis and principal component analysis showed that the MG06 and HG06 groups were more aggregated than the CK group (Figures 9B,C). It is possible, therefore, that higher concentrations (MG06 and HG06) may be required for the probiotics to be effective. In terms of species composition, at the top were *Firmicutes*, *Bacteroidota*, *Deferribacterota*, *Desulfobacterota*, *Campylobacterota,* and *Actinobacteriota* (Figure 9D); the difference in total (*Firmicutes* plus *Bacteroidota*) abundance between groups was not significant, but the *Firmicutes*/*Bacteroidota* in the LG06 group were significantly higher than those of the MG06 (*p* < 0.01) and HG06 groups (*p* < 0.05; Figure 9E). Figure 9F shows the top 10 groups with respect to the abundance of intestinal microbial genera in mice. Figure 9G shows a UNIFRAC heat map: the MG06 and HG06 groups were similar to the CK group except for a few samples (CK-9), but differed from the LG06 group. The main pathways enriched according to KEGG were carbohydrate metabolism, amino acid metabolism, etc. (Figure 9H).

### 3.5. Whole-genome sequencing and bioinformatics processing

**Figure 10:**
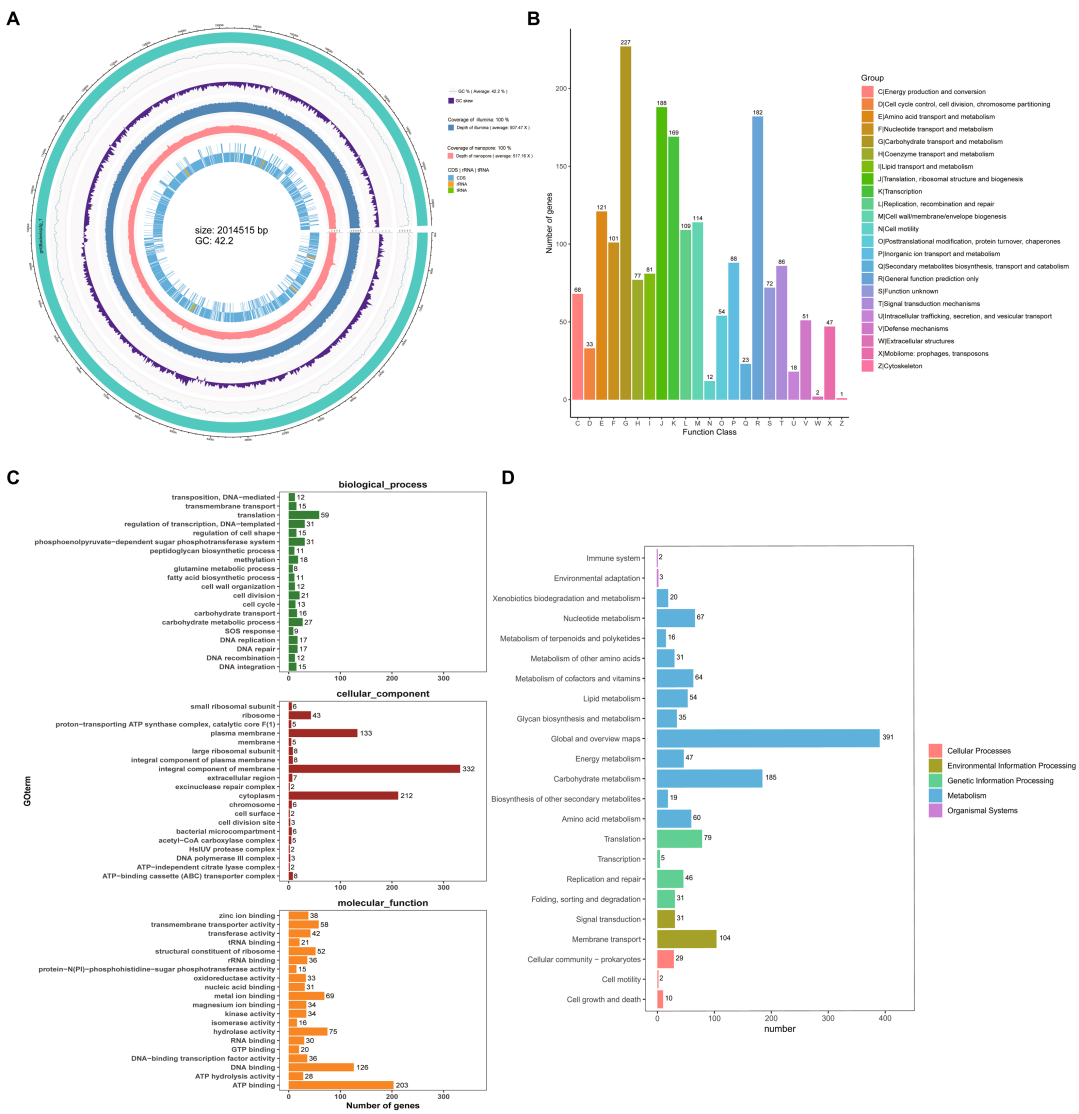
Whole-genome sequencing of GLP06. **(A)** The complete circular genome map of strain GLP06; the outermost circle of the circle diagram is genomic sequence information; the second circle is guanine and cytosine (G + C) content curve of the genomic sequence; the third circle is guanine and cytosine skew curve of the genomic sequence; the fourth circle is the second-generation sequencing depth and coverage information; the fifth circle is third-generation sequencing depth and coverage information; the sixth circle is the coding sequence and the non-coding RNA regions (rRNA, tRNA) in the reference genome, represented as inner and outer layers, with the outer layer representing positive strands and the inner layer representing negative strands. **(B)** The COG of proteins functional classification of the GLP06 strain genome; **(C)** GO analysis of strain GLP06 genome; **(D)** KEGG pathways enrichment for strain GLP06 genome.

To understand the properties of the probiotics and explore the potential of GLP06, we performed whole-genome sequencing. The complete circular genome map of GLP06 is shown in Figure 10A. The complete genome of GLP06 comprises one 2.07 Mbp circular chromosome and one circular plasmid, with guanine and cytosine (G + C) contents of 42.20 and 40.09%, respectively. Table 2 shows the genomic information of GLP06; the genome contains a total of 2077 genes with an average length of 874 bp, and the total length of the gene sequences is 1,814,434 bp, accounting for 85.13% of the total genome length. CRISPR prediction of the genome using MinCED (Version: 0.4.2) showed that the genome of GLP06 contains eight CRISPRs (Supplementary Table S8) and did not predict any drug resistance genes. The KEGG, COG and GO databases were used to analysis the gene functions of *P. acidilactici* GLP06 (Figures 10B–D).

**Table 2:** General genomic information of the strain GLP06.

Indicator	GLP06
Total raw reads number	7,190,280
Total raw bases	1,078,542,000
Clean reads	7,157,352
Clean bases	1,068,369,727
G + C content of chromosome (%)	42.20
G + C content of plasmid (%)	40.09
Genome	2,069,888
Gene number	2,077
Gene total length (bp)	1,814,434
Gene average length (bp)	874
Gene/ Genome (%)	87.66
Number of coding sequences	1,976
tRNA	58
23S rRNA	5
16S rRNA	5
5S rRNA	5
tmRNA	1

## 4. Discussion

There has been a growing interest in using microorganisms as probiotics in recent years, with LAB being the most commonly used (El-Naggar, 2004). As it is essential to introduce microorganisms that do not alter the resident microbiota, probiotics should usually be isolated and identified from a homologous host so that they can be better adapted to the GIT and enhance the benefits of probiotic agents (Dunne et al., 2001; Argyri et al., 2013). Early reports on probiotics for companion animals focused on *Lactobacillus*, *Bifidobacterium* and *Enterococcus* (Weese and Martin, 2011; Schmitz and Suchodolski, 2016; Jugan et al., 2017; Sanders et al., 2019). However, there are few studies on canine derived *Pediococcus* spp. In this study, *P. acidilactici* GLP06 was isolated and identified from the feces of beagles and evaluated for its probiotic and safety properties. In addition, whole-genome sequencing of the strain was carried out to explore its potential biological functions. This study provided a theoretical basis for using canine derived probiotics as functional pet food.

LAB have been reported to be promising alternatives to antibiotics against pathogens (Özogul and Hamed, 2018). In this study, we selected several pathogenic bacteria reported to be associated with certain gastrointestinal diseases, including gastrointestinal infections, such as *E. coli*, *S. aureus*, *S. typhimurium*, *L. monocytogenes* and *P. aeruginosa* (Vesterlund et al., 2006). Among all isolated strains, GLP02 and GLP06 showed the highest zones of inhibition against these pathogens, indicating that the two strains have excellent antibacterial activity. In addition, BS and CFS showed more potent antimicrobial activity than BP. In earlier studies, it was reported that antimicrobial activity was related to competitive exclusion mechanisms *in vivo*, where probiotics competed with pathogens for attachment sites and nutrients, preventing pathogen colonization (Lee et al., 2014). LAB can produce a variety of antimicrobial metabolites, such as organic acids, bacteriocins, hydrogen peroxide, and inhibitory enzymes, to combat pathogenic bacteria (Hasannejad Bibalan et al., 2017; Cui et al., 2018; Cervantes-Elizarrarás et al., 2019). In this study, the isolates completely lost their inhibitory effect on pathogenic bacteria after the pH of the CFS was adjusted to 7.0 (CFS_PH 7.0_), implying that the inhibitory effect of the strain may be due to the organic acids produced (Arena et al., 2016). However, further research is needed to investigate the mechanism of this bacterial inhibition.

One of the crucial characteristics in selecting a probiotic strain that is beneficial to the host is resistance to different gastrointestinal conditions, including low pH, bile salts, artificial gastrointestinal fluids (various digestive enzymes), and other conditions (Ferrari Ida et al., 2016). Many studies have evaluated the resistance of *P. acidilactici* from different sources to gastrointestinal conditions (Barbosa et al., 2015; Noohi et al., 2016). In this study, GLP02 and GLP06 showed reasonable survival rates with regard to resistance to acid, bile salts, and gastrointestinal models. Probiotic products mostly require a spray drying process (high temperature) during processing (Simpson et al., 2005; Tafti et al., 2013). Therefore, screening strains for good thermal stability would have an industrial advantage (De Angelis et al., 2006). In this study, GLP06 showed good tolerance to high temperatures. Hemolysis assays and antibiotic resistance are crucial indicators of the safety of probiotics. Many earlier studies reported that probiotic bacteria did not show hemolytic activity (Bujnakova and Strakova, 2017; Nami et al., 2018; Mangia et al., 2019; Tarrah et al., 2019). In this study, neither strain showed hemolytic activity and this indicated that these bacteria were non-toxic. Therefore, these two strains may be candidates for safe probiotics (Li et al., 2020; Rajput et al., 2022). We also explored the identification of physiological and biochemical properties of GLP02 and GLP06 strains and the results are presented in Supplementary Table S9.

Earlier studies have reported that most probiotic bacteria are resistant to aminoglycoside antibiotics (Temmerman et al., 2003; Zhou et al., 2005), which is consistent with the results of the present study. However, LAB has previously been reported to be sensitive to β-lactam antibiotics (Liasi et al., 2009), which contradicts the current study’s findings. This difference could be due to a faulty cell wall autolysis system (Perreten et al., 2001). Antimicrobial resistance genes are harmful to the host and need to be evaluated in probiotic screening (Llor and Bjerrum, 2014). Ideally, probiotics should be sensitive to at least two antibiotics or not carry intrinsic antimicrobial resistance genes, to reduce the risk of transmission (Borriello et al., 2003; Adetoye et al., 2018). In this study, the target protein sequences were annotated using a blast-based CARD database based on GLP06 whole-genome sequencing data, and no resistance genes were annotated in GLP06, indicating that strain GLP06 is safe as a potential probiotic. The surface hydrophobicity and self-agglutination properties of bacteria facilitate their adhesion to host cell surfaces and penetration into host tissues (Rodrigues and Elimelech, 2009; Heilmann, 2011), which are important indicators for evaluating probiotic function. At the same time, adhesion of a probiotic bacterial strain to the intestinal epithelium must be evaluated (Ouwehand et al., 1999; Saarela et al., 2000); adhesion can reflect the strain’s time in the host and is a prerequisite for subsequent probiotic function (Juntunen et al., 2001). In the present study, a cell line was employed that has been widely used as a model for studying the intestinal barrier *in vitro* (Dimitrov et al., 2014). In this study, the hydrophobicity, self-agglutination properties, and adhesion to Caco-2 cells of GLP06 were higher than those of GLP02, indicating that GLP06 is more suitable as a potential canine derived probiotic.

Through a series of *in vitro* tests, GLP06 showed better probiotic potential and was selected for *in vivo* safety evaluation. Mice are the most commonly used models to study host-microbiome functions and mechanisms because the mouse model allows a high level of control and improved experimental reproducibility (Nguyen et al., 2015). The evaluation showed that supplementation with the isolate had no adverse effects on growth performance or organ index in mice. A simultaneous infusion of 10^10^ CFU/mL (MG06) of the probiotic improved growth performance. The thymus coefficient, a marker of immune system development (Gao et al., 2018; Nabukeera-Barungi et al., 2019), was significantly increased in the LG06 and MG06 groups. This finding suggests that strain GLP06 may have a healthy and beneficial effect on animals (Li et al., 2020). This experiment used instillation of probiotics in the mice, which could cause irritation or toxicity. Therefore, mice were tested for indicators of liver function (AST, ALT, T-BIL, I-BIL and D-BIL) and kidney function (BUN), as well as indicators of (MDA) and antioxidant capacity (SOD). SOD protects organisms from oxidative damage by converting superoxide radicals into hydrogen peroxide, which is then degraded into water and oxygen (Yao et al., 2005). MDA can induce cellular damage in various ways, and its levels in mice reflect the levels of free radicals produced by lipid peroxidation. Serum SOD levels were significantly increased in the HG06 and MG06 groups, and serum MDA and BUN levels were significantly decreased in the MG06 group. These results indicated that GLP06 supplementation not only had no toxic effects on mice but also played a vital role in promoting immune system development and reducing oxidative stress. The *in vitro* free radical scavenging assay (DPPH and ABTS) of the GLP06 strain showed the same results.

Although our data suggested that probiotics improved health in mice, we next investigated whether probiotics affect the gut microbiota that may regulate host health. Canine-derived probiotics have been reported to have many beneficial effects on the gastrointestinal microbiomes and immune systems of a variety of species (O’Mahony et al., 2009; Herstad et al., 2022). The richness, Chao1, Shannon, Simpson, PD whole tree, and ACE indices were used to assess species richness and diversity. With the exception of the Shannon and Simpson indices, the diversity indices in the probiotic-fed group tended to be higher than those in the control group and significantly higher in the high-dose addition group; these values suggested that the probiotic-added group had higher levels of bacterial biodiversity and community diversity. At the species composition phylum level, we found an increase in the abundance of *Firmicutes* in the probiotic-added group and an increase in the combined abundance of *Firmicutes* and *Bacteroidota* in the MG06 group, but the differences between the groups were not significant. Interestingly, *Firmicutes* abundance seemed to be inversely proportional to *Bacteroidota* abundance, which may indicate that they occupy the same ecological niche (Vázquez-Baeza et al., 2016). At the genus level, *Lactobacillus* and *Muribaculaceae* abundance was significantly higher in the MG06 group. The addition of canine-derived LAB increased *Lactobacillus* abundance, as reported in other studies (Baillon et al., 2004; Gagné et al., 2013). The main fermentation product of *Muribaculaceae* is propionate, which is associated with intestinal health and extended lifespan in mice (Smith et al., 2021). Studies using culture-free methods have shown that *Muribaculaceae* specializes in fermentation of complex polysaccharides (Ormerod et al., 2016; Lagkouvardos et al., 2019). *Muribaculaceae* and *Clostridium perfringens* are the main mucin monosaccharide foragers, occupying the same ecological niche in the gut. Increased *Muribaculaceae* will digest N-acetylglucosamine and hinder the colonization of the gut by *Clostridium perfringens* (Hiraishi et al., 2022). This finding suggests that the GLP06 probiotic may inhibit *Clostridium perfringens* and increase *Muribaculaceae* enrichment to improve intestinal health in mice. One study fed *Pediococcus pentosaceus* CECT 8330 to mice with colitis and found that the strain reduced levels of proinflammatory cytokines (TNF-α, IL-1β, and IL-6), and increased levels of IL-10 and abundance of *Muribaculaceae* and *Lactobacillus*; the authors concluded that *P. pentosaceus* CECT 8330 could be a promising probiotic to reduce intestinal inflammation (Dong et al., 2022). A limitation of the results was that *Muribaculaceae* was specific to the murine intestine, and experiments in dogs are needed to explore the role of *P. acidilactici* GLP06. In spite of its limitations, this study added to our understanding of the effect of *P. acidilactici* GLP06 on host intestinal flora.

In this study, the whole genome of *P. acidilactici* GLP06 was sequenced to elucidate its potential biological functions. The genome size of *P. acidilactici* GLP06 isolated in this study was 2,014,515 bp, a medium-sized genome, and these bacteria are usually highly metabolizable, tolerant and well adapted (Ranea et al., 2004). Based on GO, KEGG, and COG annotation results, we identified genes in global and overview maps involved in carbohydrate metabolism, membrane transport, translation, and nucleotide metabolism. Interestingly, the gene analysis revealed many common carbohydrate metabolism-related genes in *P. acidilactici* strains. We conjecture that the carbohydrate metabolism-related genes of canine-derived strains are closely related to the domestication of canines. Earlier studies reported whole-genome resequencing of dogs and wolves to screen for candidate mutations in genes critical to canine domestication and to provide functional support for increased starch digestion in dogs relative to wolves, a critical step in canine domestication (Axelsson et al., 2013). Dogs have lived with and in a similar environment to humans for long periods after domestication, and the gut microbiomes of canines and humans are relatively similar, with prolonged dietary alterations affecting the composition of the canine gut microflora (Coelho et al., 2018). We hypothesize that the carbohydrate-related genes in the GLP06 genome are the result of long-term evolution and domestication in dogs. We also enriched carbohydrate metabolism in previously predicted results for mouse intestinal flora KEGG, suggesting that strain GLP06 may modulate the host intestinal flora to improve health by regulating carbohydrate metabolism. Furthermore, the genome of GLP06 contains CRISPR, which has been identified in other probiotic studies (Alayande et al., 2020; Pan et al., 2020). CRISPRs can limit the spread of antimicrobial resistance genes and provide the potential for defense against incoming extrachromosomal DNA molecules (Marraffini et al., 2006; Mohanan et al., 2012). This finding suggests that the *P. acidilactici* GLP06 genome is stable and may be a candidate for companion animal probiotics.

## 5. Conclusion

In conclusion, in this study, a strain of *P. acidilactici* GLP06 was isolated from the feces of beagles. It showed good resistance to gastrointestinal pathogenic bacteria, good tolerance to the gastrointestinal environment and heat, resistance to aminoglycoside antibiotics but sensitivity to β-lactam antibiotics (Piperacillin and Imipenem), γ-hemolysis, high cell surface hydrophobicity, strong self-aggregation, and good adhesion to Caco-2 cells, indicating that *P. acidilactici* GLP06 had excellent probiotic properties and an ideal safety profile. *In vivo* experiments showed that *P. acidilactici* GLP06 supplementation not only had no toxic effects on mice but also promoted the development of the immune system, improved resistance to oxidative stress, and increased the diversity of intestinal flora and the abundance of *Lactobacillus* at suitable concentrations (MG06 group). Whole-genome sequencing showed that *P. acidilactici* GLP06 had one chromosome and one plasmid containing 1,976 coding sequences, representing 86.12% of the genes, with no resistance genes and eight CRISPR sequences, indicating that the strain’s genome was stable and free from the risk of resistance gene transfer. However, it is necessary to further reveal its specific health benefits through *in vivo* experiments in dogs. In conclusion, *P. acidilactici* GLP06 is a promising candidate probiotic with potential future use in the companion animal health and food industries.

## Data availability statement

The datasets presented in this study can be found in online repositories. The names of the repository/repositories and accession number(s) can be found in the article/Supplementary material.

## Ethics statement

The animal study was reviewed and approved by Qingdao Agricultural University, Animal Science and Technology College (protocol code DKY2022003 and 2022.06.10).

## Author contributions

GL: conceptualization, writing review and editing, supervision, project administration, and funding acquisition. MZ, KL, YZ, and YL: methodology. MZ, YL, and YZ: software and formal analysis. MZ and KL: data curation. MZ: writing–original draft preparation. All authors have read and agreed to the published version of the manuscript.

## Funding

This study was funded by the Start-up Fund for Scientific Research of High-level Talents of Qingdao Agricultural University (1121021) and the Functional pet food research and development project (20220122) of Shandong Chongzhiyoupin Pet Food Co., Ltd. to GL the funder was not involved in the study design, collection, analysis, interpretation of data, the writing of this article, or the decision to submit it for publication.

## Conflict of interest

NZ was employed by Shandong Chongzhiyoupin Pet Food Co., Ltd.

The remaining authors declare that the research was conducted in the absence of any commercial or financial relationships that could be construed as a potential conflict of interest.

## Publisher’s note

All claims expressed in this article are solely those of the authors and do not necessarily represent those of their affiliated organizations, or those of the publisher, the editors and the reviewers. Any product that may be evaluated in this article, or claim that may be made by its manufacturer, is not guaranteed or endorsed by the publisher.

## Footnotes

^1^ https://github.com/rrwick/Unicycler

^2^ https://github.com/ctSkennerton/minced/tree/master
