# Corrigendum: Probiotic characteristics and whole-genome sequence analysis of *Pediococcus acidilactici* isolated from the feces of adult beagles

**DOI:** 10.3389/fmicb.2023.1235657

**Published:** 2023-07-10

**Authors:** Mengdi Zhao, Keyuan Liu, Yuanyuan Zhang, Yueyao Li, Ning Zhou, Guangyu Li

**Affiliations:** ^1^College of Animal Science and Technology, Jilin Agricultural University, Changchun, China; ^2^College of Animal Science and Technology, Qingdao Agricultural University, Qingdao, China; ^3^Shandong Chongzhiyoupin Pet Food Co., Ltd., Weifang, China

**Keywords:** beagles, probiotic, feces, whole-genome sequence, *Pediococcus acidilactici*

In the published article, there was an error in affiliation 1. Instead of “College of Animal Science and Technology, Changchun Agricultural University, Changchun, China,” it should be “College of Animal Science and Technology, Jilin Agricultural University, Changchun, China.”

The authors apologize for this error and state that this does not change the scientific conclusions of the article in any way. The original article has been updated.

