# Supplementary material for: Probiotic characteristics and whole-genome sequence analysis of Pediococcus acidilactici isolated from the feces of adult beagles
Source: Front Microbiol. 2023 May 15;14:1179953. doi: 10.3389/fmicb.2023.1179953 (PMC10225567; doi:10.3389/fmicb.2023.1179953)
Supplement: Supplementary file 1 [file Data_Sheet_1.docx]

Supplementary Material

Probiotic Characteristics and Whole Genome Sequence Analysis of *Pediococcus acidilactici* Isolated from Faecal of Adult Beagles

**Mengdi Zhao^1,2^, Keyuan Liu^2^, Yuanyuan Zhang^2^, Yueyao Li^2^, Ning Zhou^3^ and Guangyu Li ^2^***

*** Correspondence:** Guangyu Li：[tcslgy@126.com](mailto:tcslgy@126.com)

**The 16S sequence in this study is deposited in the NCBI at GenBank with the accession numbers OQ449392-OQ449393; the whole genome sequence data deposit is accession number PRJNA935859 (https://www.ncbi.nlm.nih.gov/bioproject/PRJNA935859) (21- Feb -2022).**

**S1.** Morphology and Gram stain results of GLP02 and GLP06. (A) Morphology results of GLP02; (B) Gram stain results of GLP02; (C) Morphology results of GLP06; and (D) Gram stain results of GLP02.


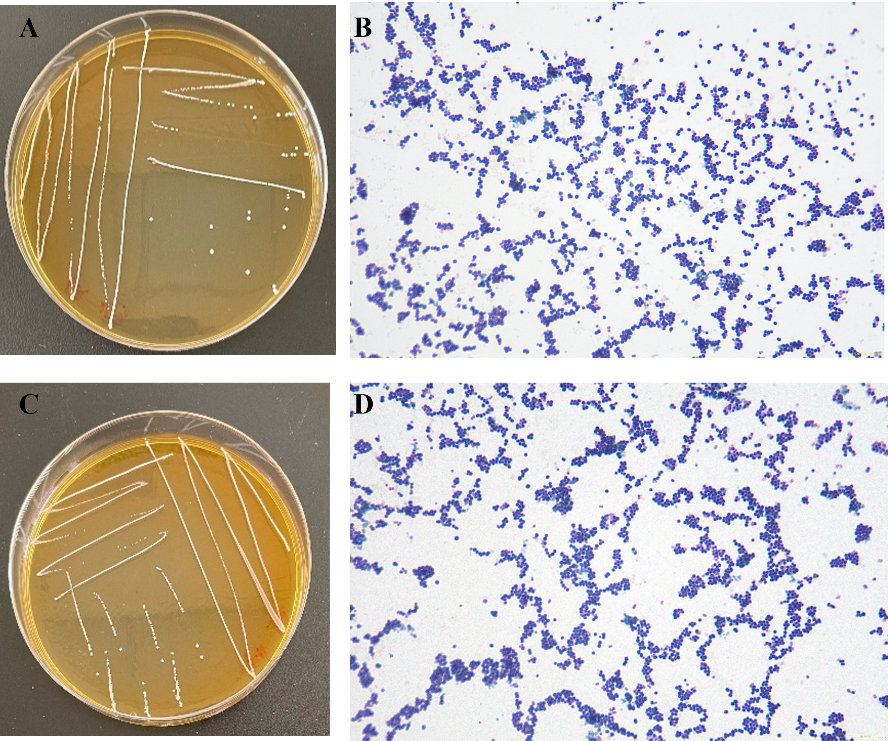


**S2**. The inhibitory effects of isolated strains against pathogenic indicator bacteria. The pathogenic indicator bacteria were (A) *E. coli*; (B) *Salmonella*; (C) *S. aureus*; (D) *L. monocytogenes*; and (E) *P. aeruginosa*. In each LB agar plate, a) was added to the bacterial precipitate of GLP02; b) was added to the bacterial precipitate of GLP06; c) was added to the MRS broth; d) was added to the cell-free supernatant pH_7.0_ of GLP02 and e) was added to the cell-free supernatant pH_7.0_ of GLP06.


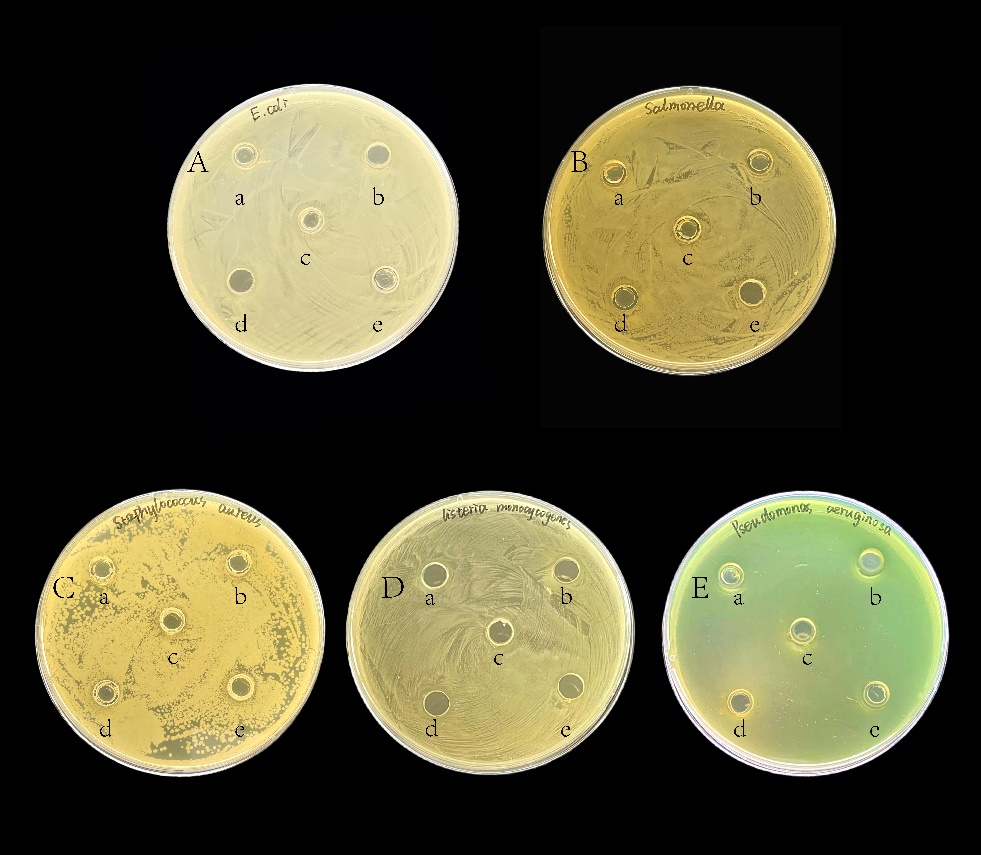


| Items | *E. coli* | *Salmonella* | *S. aureus* | *P. aeruginosa* | *L.monocytogenes* |
| --- | --- | --- | --- | --- | --- |
| CFS GLP02 | 12.11±0.27^a^ | 13.27±0.29^a^ | 11.41±0.08^a^ | 12.12±0.20^a^ | 15.60±0.16^b^ |
| CFS GLP06 | 14.09±0.41^b^ | 12.75±0.44^a^ | 14.50±0.08^b^ | 13.79±0.25^b^ | 13.78±0.33^a^ |
| BS GLP02 | 17.88±0.15^c^ | 19.03±0.04^c^ | 17.86±0.51^c^ | 17.91±0.19^c^ | 21.68±0.04^c^ |
| BS GLP06 | 18.13±0.39^c^ | 16.92±0.95^b^ | 18.27±0.27^c^ | 17.95±0.14^c^ | 21.42±0.61^c^ |
| BP GLP02 | - | - | - | - | - |
| BPGLP06 | - | - | - | - | - |
| CFSpH_7.0_ GLP02 | - | - | - | - | - |
| CFSpH_7.0_ GLP06 | - | - | - | - | - |
| MRS broth | - | - | - | - | - |

Values are displayed as the mean ± SD. *p* < 0.05 indicates differences among the CFS and BS ,“–” indicates no antibacterial effect.

**S3.** Statistics of GLP06 plasmid annotation results.

| Item | Accession | Identity (%) | E-value | Taxon |
| --- | --- | --- | --- | --- |
| GLP06 | NZ_CP028250.1 | 99.974 | 0 | *Pediococcus acidilactici* strain SRCM102732 |

**S4**. Results of the Hemolytic Activity for GLP02 and GLP06. (A) Results of the Hemolytic Activity for GLP02; and (B) Results of the Hemolytic Activity for GLP06. Staphylococcus aureus inoculated by underlining in the lower half of figure (A) and (B), used as a positive control.


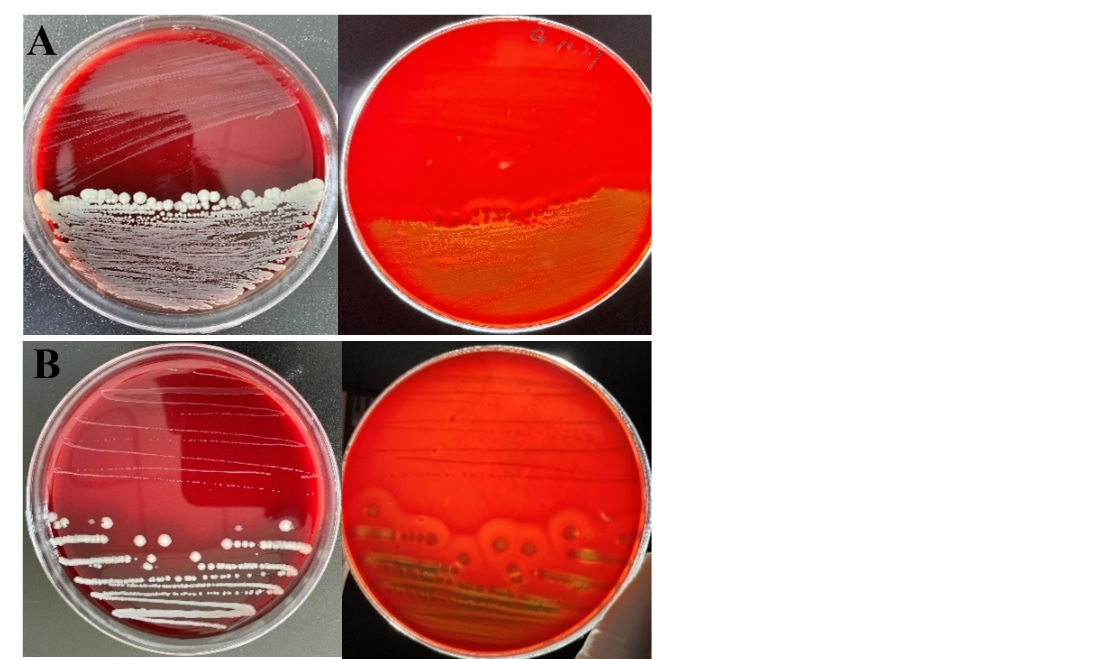


**S5.** Antibiotic resistance of strain GLP02.

| Antimicrobial classes | Antimicrobial agents | Disk dose (μg) | Inhibition zone diameters/mm (IZD) ^a^ | | |
| --- | --- | --- | --- | --- | --- |
|  |  |  | ≤15 mm (R) | 16–20 mm (I) | ≥21 mm (S) |
| β-lactams  antibiotics | Penicillin | 10 | X ^R^ |  |  |
|  | Oxacillin | 1 | X ^R^ |  |  |
|  | Ampicillin | 10 | X ^R^ |  |  |
|  | Piperacillin | 100 |  |  | 23.02±0.75 ^S^ |
|  | Imipenem | 10 |  |  | 22.12±2.12 ^S^ |
| Glycopeptides | Vancomycin | 30 | X ^R^ |  |  |
| Aminoglycosides  antibiotics | Streptomycin | 10 | X ^R^ |  |  |
|  | Gentamicin | 10 | X ^R^ |  |  |
|  | Amikacin | 30 | X ^R^ |  |  |
|  | Kanamycin | 30 | X ^R^ |  |  |
| Broad-spectrum  antibiotics | Tetracycline | 30 | X ^R^ |  |  |
|  | Chloramphenicol | 30 |  |  | 22.14±2.99 ^S^ |
|  | Minocycline | 30 |  | 15.13±5.50 ^I^ |  |
|  | Doxycycline | 30 |  | 20.14±2.08 ^I^ |  |
|  | Cotrimoxazole | 25 | X ^R^ |  |  |
| Macrolides | Azithromycin | 15 | X ^R^ |  |  |
|  | Erythromycin | 15 |  | 18.67±1.65 ^I^ |  |
|  | Clindamycin | 2 |  |  | 23.63±3.51 ^S^ |
| Fluoroquinolone  antibiotics | Norfloxacin | 10 | X ^R^ |  |  |
|  | Ciprofloxacin | 5 | X ^R^ |  |  |
|  | Levofloxacin | 5 | X ^R^ |  |  |

**S6.** Effect of *GLP06* supplementation on organ coefficients in mice. The (A) heart coefficient;(B) liver coefficient;(C) spleen coefficient and (D) kidney coefficient were calculated as heart / liver / spleen / kidney weigh/body weigh × 100. Values were displayed as the mean ± SD, n=10.


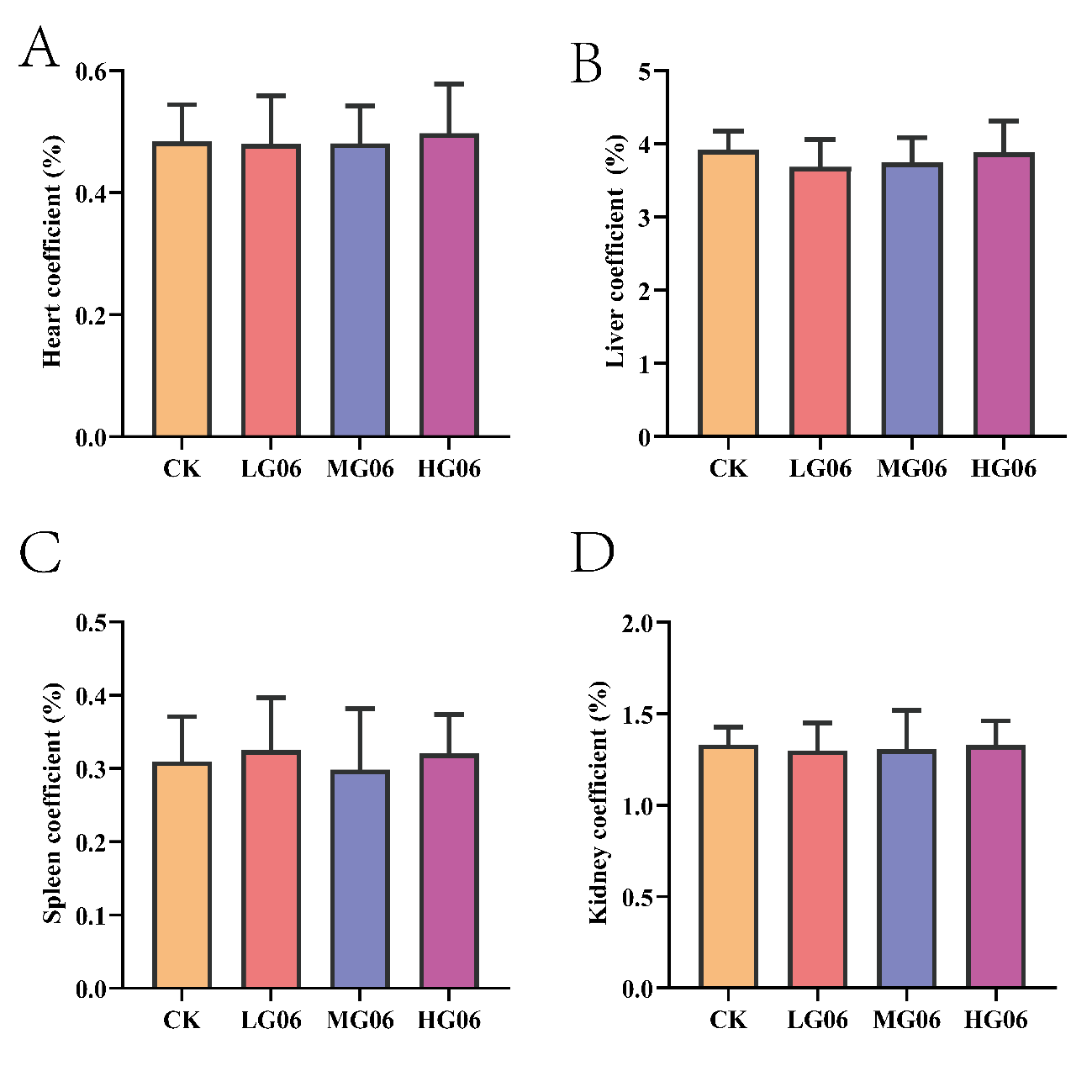


**S7.** Effect of *GLP06* supplementation on serum biochemical parameters on in mice. The levels of serum (A) AST; (B) ALT; (C) T-BIL;(D) I-BIL and (E) D-BIL were determined by use of commercial ELISA kits. Values are displayed as the mean ± SD, n=10.


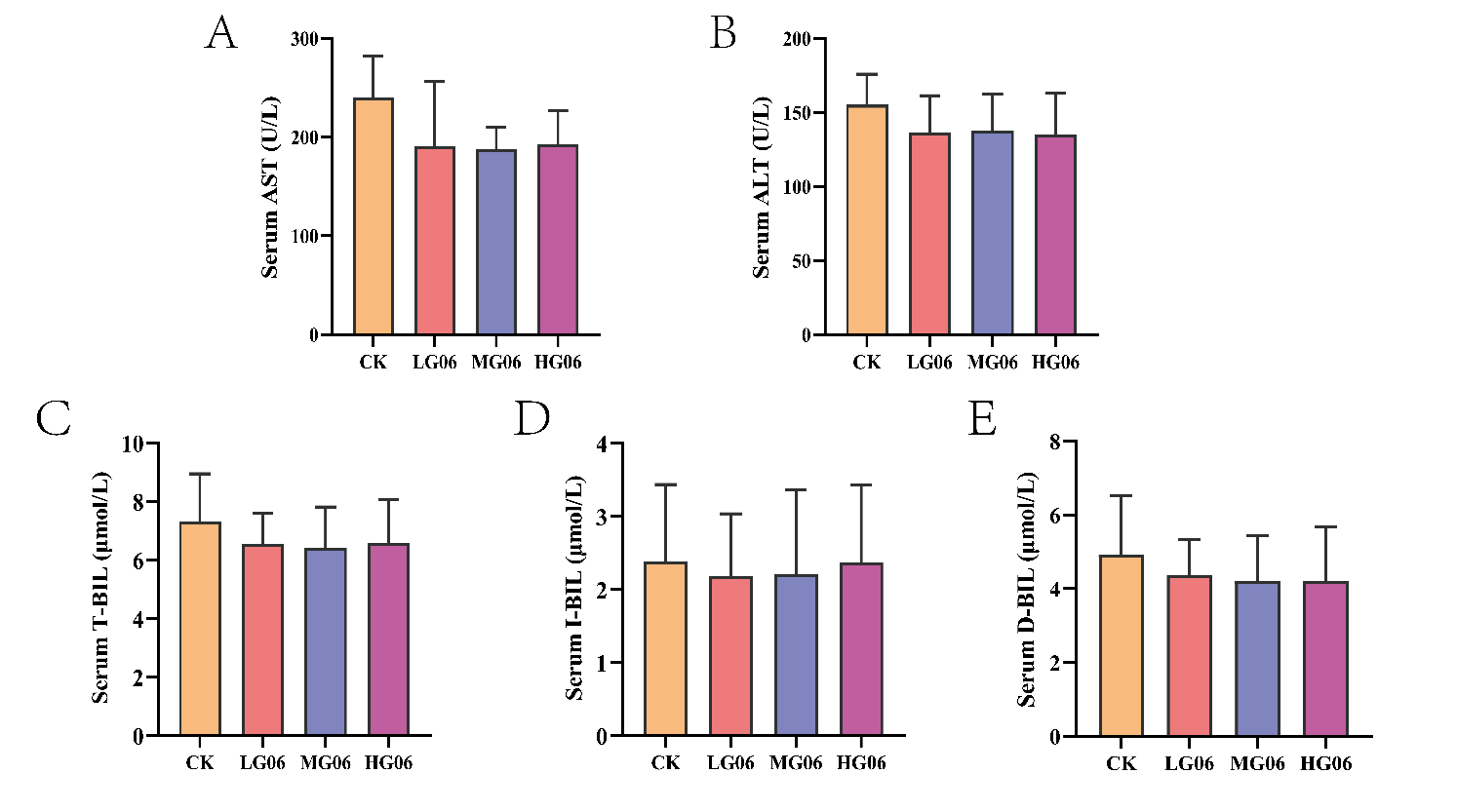


**S8**. CRISPR prediction result statistics of the strain GLP06.

| ID | Start position | End  position | Score | Repeat unit sequence |
| --- | --- | --- | --- | --- |
| CRISPR1 | 53206 | 54190 | 12 | ATGATATCGCTAGTGAGTCAGCATCGCG |
| CRISPR2 | 54234 | 54349 | 2 | GCATCTGACCATGAAAGTGAATCAATTTC |
| CRISPR3 | 54606 | 54997 | 5 | TCACAAGATGTAAATAGTGAATCGGTATCCCGTTCAACATCGGC |
| CRISPR4 | 55684 | 55951 | 4 | CATCTGACCACGAAAGTGAATCAATTTCAATGTCTAA |
| CRISPR5 | 102468 | 102785 | 5 | GAAGAATGATCATCACCAGATGAACTA |
| CRISPR6 | 456077 | 456178 | 2 | GTTTCAGAAGGATGTTAAATCAATAAGGTTAAGATC |
| CRISPR7 | 701890 | 702070 | 3 | GGCTCAGGCTCTGACTCGAACAACG |
| CRISPR8 | 1165903 | 1166048 | 3 | CCAATGTCTTGGTAGAAATCCTTGTC |

**S9**. Identification of physiological and biochemical characteristics of strain GLP02 and GLP06.

| Items | Results ^a^ | | Items | Results ^a^ | |
| --- | --- | --- | --- | --- | --- |
|  | GLP02 | GLP06 |  | GLP02 | GLP06 |
| Aesculin | - | - | Mannitol | - | - |
| Glucose | + | + | Sorbitol | - | - |
| Cellobiose | + | + | Starch hydrolysis | - | - |
| Galactose | + | + | Nitrate (gas production) | - | - |
| Maltose | + | + | Hydrogen sulfide | - | - |
| Rhamnose | - | - | Indole test | - | - |
| Salicin | - | - | Nitrate reduction | - | - |
| Sucrose | - | - | Voges-Prokauer test | - | - |
| L-Rhamnose | - | - | Methyl Red test | + | + |
| Lactose | - | - | Gelatin liquidized | - | - |
| Raffinose | - | - | Hydrogen peroxide | - | - |
| Fructose | + | + | Mobility | - | - |
| Pectinose | + | + | Xylose | + | + |
| Inulin | - | - | Urea | - | - |
| 1% sodium equine | - | - | Oxidase | - | - |

^a^. Symbols＋: positive；-: negative.
