# Supplementary figures and images for: Probiotic characteristics and whole-genome sequence analysis of Pediococcus acidilactici isolated from the feces of adult beagles
Source: Front Microbiol. 2023 May 15;14:1179953. doi: 10.3389/fmicb.2023.1179953 (PMC10225567; doi:10.3389/fmicb.2023.1179953)

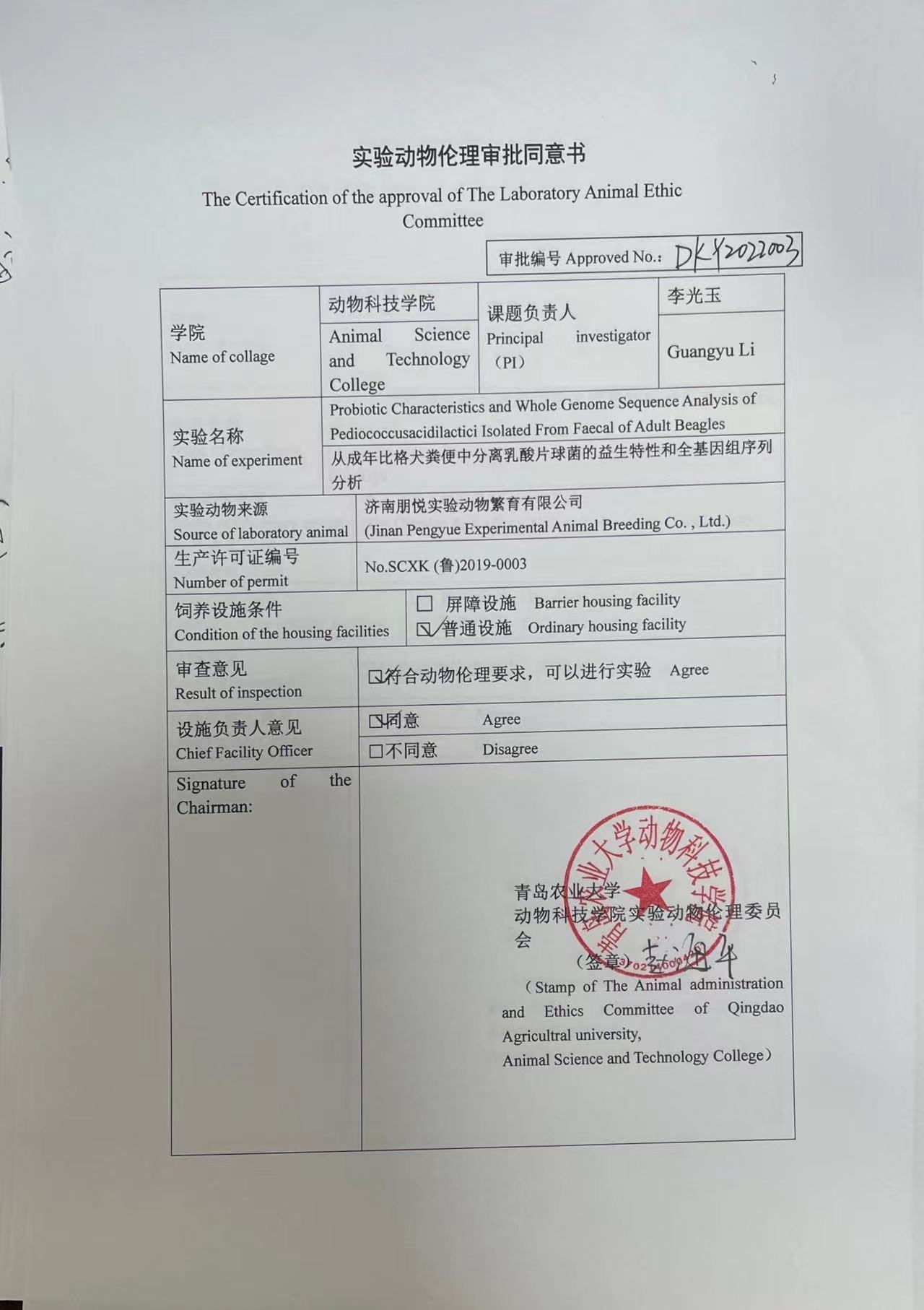

Supplement: Supplementary file 2 [file Image_1.JPEG]
